# Catechol thwarts virulent dimorphism in *Candida albicans* and potentiates the antifungal efficacy of azoles and polyenes

**DOI:** 10.1038/s41598-021-00485-2

**Published:** 2021-10-26

**Authors:** Ravi Jothi, Ravichellam Sangavi, Ponnuchamy Kumar, Shunmugiah Karutha Pandian, Shanmugaraj Gowrishankar

**Affiliations:** 1 Department of Biotechnology, Science Campus, Alagappa University, Karaikudi, Tamil Nadu 630 003 India; 2 Food Chemistry and Molecular Cancer Biology Lab, Department of Animal Health and Management, Alagappa University, Karaikudi, India

**Keywords:** Biofilms, Fungi, Microbiology

## Abstract

The present study was deliberately focused to explore the antivirulence efficacy of a plant allelochemical—catechol against *Candida albicans*, and attempts were made to elucidate the underlying mechanisms as well. Catechol at its sub-MIC concentrations (2–256 μg/mL) exhibited a dose dependent biofilm as well as hyphal inhibitory efficacies, which were ascertained through both light and fluorescence microscopic analyses. Further, sub-MICs of catechol displayed remarkable antivirulence efficacy, as it substantially inhibited *C. albicans’* virulence enzymes i.e. secreted hydrolases. Notably, FTIR analysis divulged the potency of catechol in effective loosening of *C. albicans*’ exopolymeric matrix, which was further reinforced using EPS quantification assay. Although, catechol at BIC (256 μg/mL) did not disrupt the mature biofilms of *C. albicans*, their initial adherence was significantly impeded by reducing their hydrophobic nature. Besides, FTIR analysis also unveiled the ability of catechol in enhancing the production of farnesol—a metabolite of *C. albicans*, whose accumulation naturally blocks yeast-hyphal transition. The qPCR data showed significant down-regulation of candidate genes viz., *RAS1, HWP1* and *ALS3* which are the key targets of Ras-cAMP-PKA pathway -the pathway that contribute for *C. albicans*’ pathogenesis. Interestingly, the up-regulation of *TUP1* (a gene responsible for farnesol-mediated hyphal inhibition) during catechol exposure strengthen the speculation of catechol triggered farnesol-mediated hyphal inhibition. Furthermore, catechol profusely enhanced the fungicidal efficacy of certain known antifungal agent’s viz., azoles (ketoconazole and miconazole) and polyenes (amphotericin-B and nystatin).

## Introduction

Over the past few decades, the prevalence of invasive fungal infections caused by *Candida* species has been persistently increasing especially in immunosuppressed individuals^1,2^. Globally, most individuals encounter curable superficial fungal infections at least once in their lifetime. However, millions of people suffer from life-threatening invasive fungal infection, which is much harder to diagnose and treat^3^. The global incidence of candidemia has been estimated at 6.0–13.3 cases per 100,000 population with an associated mortality rate of 50%, which is predicted to be even higher than the death rate caused by tuberculosis or malaria^4^. It has been reported that about 80% of nosocomial fungal infections are caused by *Candida* species. Amongst, *Candida albicans*, *Candida tropicalis* and *Candida glabrata* were found to be the major players^5^. In the midst, *C. albicans* is by far the most prevalent etiological agent of invasive candidiasis and are often ranked top in the list of most common fungal pathogens, causing severe disseminated infections in humans^6^. In United States, candidemia account for nearly 9% of nosocomial infections, of which 40–70% have been caused by *C. albicans*^7^. *C. albicans* being a diploid microorganism, it exists either in the form of yeast or hyphae, and reside as commensal in half of the healthy human population where it is controlled by the host and microbial interactions^8^. On the other hand, they can be able to cause irreversible pathogenic effect ranging from superficial to life-threatening systemic infections through its intrinsic yeast-to-hyphal transforming ability^9^.

*C. albicans* establish infection through four stages viz., colonization, superficial, deep-seated and systemic^10^. In the colonization stage, the growth of *C. albicans* is balanced by the host immune system and microflora. Nevertheless, the other three stages occur only under opportunistic circumstances promoting the overgrowth of *C. albicans*, which culminate in microflora imbalance and weakened immunity^11^. Yeast form of *C. albicans* plays an inevitable role in the process of colonization as it assists in both tissue penetration and host immune evasion. However, the hyphal form is indispensable for the pathogenesis of *C. albicans*, as it encodes several virulence genes such as secretion of adhesin, hydrolases and protease leading to host tissue damage^2^. Hence, the yeast to hyphal transition has been considered as a crucial virulence determinant of *C. albicans*. A mounting body of mutation studies reinforced that *C. albicans* mutants lacking yeast to hyphal transition displayed avirulent properties in in vivo mouse models^12,13^. Apart from morphological plasticity, myriad of factors including metabolic adaptability (ability to survive in a broad range of pH), biofilm formation, contact sensing and thigmotropism contributes to the pathogenesis of *C. albicans*^14^. At present, the therapeutic choices of antifungal agents are mostly restricted to azoles, polyenes and echinocandins. Unfortunately, these treatment choices have become unsatisfactory owing to the increased development of resistance, selective pressure, non-availability of conventional antifungal for systemic administration and adverse effects at higher concentrations^15^. Thus, there is an urgent need to develop new agents with low toxicity, broad spectrum of activity and a new mode of action against drug-resistant *C. albicans*. In order to address the above-mentioned scientific issues, therapies that specifically target virulence rather than harming the fungal metabolism have been proved to be even more promising. One such remarkable approach is the antivirulence therapy, wherein biomolecules with proficiency to exclusively hamper the virulence traits of pathogens have been envisaged with the certainty that it would nullify the phenomenon of resistance by counteracting selection pressure^16^. In this milieu, plant-derived molecules have been attaining a great deal of attention to be an ideal antivirulence agent due to their drug-like properties^17^. Lately, allelochemicals have been emphasized as a natural antivirulent owing to their short half-life i.e. rapid biodegradability^18,19^.

Catechol (C_6_H_6_O_2_), a benzenediol comprises of benzene ring at center coupled with two hydroxy substituents. It is one of the natural allelochemicals synthesized by plants especially in onions through the shikimate pathway^20,21^. Hitherto, a plethora of investigations have documented the multifaceted pharmaceutical properties of catechol viz., broad-spectrum antibacterial^19^, anticariogenic^21^ and antifungal^18^ efficacies. In addition, some reports have been revealed that catechol act as a stimulator for the root hair elongation in rice through reactive oxygen species mediated redox signaling^19^. Given the prominence of myriad therapeutic potentials of catechol, the compounds with catechol groups have been deployed in medical applications chiefly due to low cytotoxicity rendered by the holistic structure^22,23^. In fact, catechol moieties (such as dopamine and norepinephrine) found in the human body serves as essential regulators of central nervous system^24^. Thus, they are considered to be ecologically and toxicologically safer than the synthesized molecules. Nevertheless, its antivirulence propensity to impede the formation of biofilm and production of virulence in pathogenic fungi, especially *C. albicans* has not been explored till date. Therefore, the present study was consciously aimed to explore and delineate the inhibitory efficacy of catechol against biofilm assemblage, hyphal transition and secreted virulence in *C. albicans*.

## Materials and methods

### Preparation of active compound

The active compound catechol (purity ≥ 99% CAS Number: 120-80-9) was purchased from Sigma-Aldrich. The stock solution was prepared as 100 mg/mL using ethanol and stored at 4° C for further use. To determine the influence of solvent (ethanol) on *C. albicans* biofilm and growth, the medium with inoculum and ethanol was set as a vehicle control.

### Strain and culture conditions

The test organism *C. albicans* ATCC 10231 used in this study was purchased from HiMedia, India. The strain was maintained in sabouraud dextrose agar (SDA) plates and routinely cultured in yeast extract peptone dextrose (YEPD) broth at 37° C. To perform all in vitro assays, 1% of 3 h culture with 0.1 optical density (OD) (1 × 10^6^ CFU/mL) was used to inoculate in YEPD broth. Both biofilm and hyphal assays were performed in spider medium (consisting mannitol 1%, K_2_HPO_4_ 0.2% and nutrient broth 1%) to allow hyphal elongation. Glucose-phosphate-proline growth-medium (GPP) was used for the extraction of quorum sensing molecule (farnesol) from *C. albicans*.

### Evaluation of inhibitory effect of catechol on *C. albicans* planktonic growth

To ensure whether the catechol at varied range of concentrations influence the growth of *C. albicans* planktonic cells, broth microdilution assay was performed following clinical and laboratory standards institute (CLSI) 2008 standard guidelines with small alterations^25^. *C. albicans* (1 × 10^6^ CFU/mL) was dispensed into 24-well microtiter plates (MTPs) containing YEPD broth supplemented with and without catechol at various concentrations (0–1024 µg/mL) to a final volume of 1 mL/well. The YEPD medium supplemented with *C. albicans* devoid of catechol was served as a control. After incubation at 37° C for 24 h, MIC was determined by measuring the OD at 600 nm using spectrophotometer (Spectra Max 3, Molecular Devices, United States). The MIC was defined as the lowest concentration of catechol that showed visible growth inhibition in YEPD broth compared to untreated control. All experiments were carried out in triplicate.

### Alamar blue assay

Furthermore, the alamar blue assay was used to evaluate the metabolic viability of catechol treated samples as previously described by Repp et al. (2007) with minor modification^26^. Briefly, *C. albicans* cells were treated with catechol for 24 h at 37° C. The *C. albicans* without catechol treatment was acted as control. After incubation, the culture suspension was centrifuged at 8000 rpm for 10 min and the obtained pellet resuspended with sterile phosphate-buffered saline (PBS). Alamar blue (100 μg/mL) (Sigma Aldrich) was added to each cell pellets that were suspended in PBS and incubated at dark for 4–8 h. Subsequently, the fluorescent intensity of excitation (560 nm) and emission (590 nm) wavelengths were measured spectroscopically.

### Investigation of inhibitory effect of catechol on *C. albicans* biofilm

In order to examine the antibiofilm efficacy of catechol at its sub-MIC against *C. albicans* biofilm, crystal violet staining method was performed according to the protocol prescribed by Prasath et al. (2019) with required modifications^27^. Briefly, overnight culture of *C. albicans* was inoculated into 24-well MTPs having 1 mL spider medium supplemented with catechol at various concentrations (0–1024 µg/mL), and incubated at 37° C for 48 h without shaking to allow the formation of biofilm. The spider medium supplemented with *C. albicans* devoid of catechol was considered as control. After incubation, the spent medium was transferred to fresh 24-well MTPs and read at 600 nm to check the impact of catechol on *C. albicans* planktonic cells. Further, the non-adherent planktonic cells on MTP were removed by washing with sterile PBS. Then, the sessile cells on the bottom of MTP were stained for 15 min with 0.4% crystal violet (HiMedia, India). Any excess stain was removed by washing with sterile water. After 15 min of destaining with 15% glacial acetic acid, the amount of crystal violet bound to the biofilm cells was quantified spectophotometrically at 570 nm. The percentage of biofilm inhibition was determined using the following formula.

The relative biofilm inhibition: % of biofilm inhibition = [(Control OD570 nm − Treated OD570 nm)/ Control OD570 nm] × 100.

Biofilm Inhibitory concentration (BIC) was defined as the minimal concentration of catechol that brings about 90% of biofilm inhibition without affecting cellular viability.

### Microscopic visualization of *C. albicans* biofilm

In order to further ascertain the antibiofilm efficacy of catechol, various microscopic techniques viz., light and fluorescence microscopic analyses were carried out^28^. Briefly, the biofilm formation was initiated by growing *C. albicans* on 1 cm^2^ glass slide in 24-well MTPs containing spider medium with catechol at the concentration of 64, 128 and 256 µg/mL for 48 h. After incubation, non-adherent planktonic cells were removed by washing with sterile PBS and then adhered biofilms on the slides were stained accordingly with the type of the microscopic technique undergone. To envisage biofilm under the light microscope, the adhered biofilm cells on the glass slides stained for 5 min with 0.4% crystal violet. Any excess crystal violet stain was removed by washing with sterile water. After drying, the glass slides were visualized under light microscope at 200 × magnification to examine the differential architecture of formed biofilm (Nikon Eclipse 80i, Japan). To visualize biofilm architecture under fluorescence microscopy, the biofilm formed glass slides were stained by incubation at dark condition for 5 min with 0.1% acridine orange and excess dye was washed using sterile PBS. Then, the architecture of biofilm was observed under fluorescence microscopy (LSM 710, Carl Zeiss, and Germany).

### LIVE/DEAD cell viability assay

To further ascertain the non-fungicidal efficacy of catechol on *C. albicans* sessile cells, LIVE/DEAD analysis was performed using propidium iodide (PI) and acridine orange (AO) staining^27^. After 48 h treatment of *C. albicans* biofilm with catechol at concentration of 64, 128 and 256 µg/mL, 1 cm^2^ glass slide were stained using AO/PI stains (Sigma Aldrich) for 15 min at dark condition. After washing the glass pieces using phosphate buffered saline, the stained cells were visualized under fluorescence microscopy (LSM 710, Carl Zeiss, and Germany) with excitation wavelengths of 525 and 490 nm for AO and PI, respectively.

### Yeast–Hyphae (Y–H) inhibition assay in liquid media

To assess the influence of catechol on yeast to hyphal transition of *C. albicans,* hyphal growth assay in liquid media was performed as suggested by Bar-Yosef et al.^29^. In brief, an overnight culture of *C. albicans* was used to inoculate a 1 mL of spider broth supplemented with different concentrations of catechol (0–1024 µg/mL) and incubated for 24 h at 37° C. After incubation, the planktonic growth in the spent medium was measured at OD 600 nm. Then, the pellets were resuspended in 10 µL of PBS and the ratio of yeast to hyphae cells were further examined via light microscope at 200× magnifications (Nikon Eclipse 80i, Japan). Phase contrast micrographs of control and treated cells were taken using fluorescence microscopy at 400× magnifications (LSM 710, Carl Zeiss, and Germany).

### Examination of *C. albicans* colony morphology on solid media

To scrutinize the protrusion of hyphae in the presence and absence of catechol, colony morphology assay was performed on solid spider media^30^. Briefly, 5 µL of *C. albicans* culture was placed on the spider agar medium supplemented with different concentrations (64, 128 and 256 µg/mL) of catechol. Plates were then incubated for 5–6 days to allow hyphal induction. Then after, the plates were imaged using gel documentation system (GelDoc XR+, Bio-Rad, United States).

### Lipase assay

The impact of catechol on the production of lipas*e* was measured by both quantitative and qualitative methods^31^. In qualitative measurement, tributyrin agar medium consists of peptone 0.8%, yeast extract 0.4%, NaCl 0.3% and agar 1.8% was prepared by autoclaving at 121° C for 30 min. After autoclaving, tributyrin 0.2% and catechol (at 64, 128 and 256 µg/mL) were added to the medium at 43–46° C. Afterwards, 5 µL of overnight *C. albicans* culture was placed on the center of agar plate and incubated at 37° C for 2–3 days. After incubation, lipase production was examined by measuring zone of clearance around colony using Hiantibiotic zone scale (Himedia, Mumbai). For quantitative assessment, P-Nitrophenyl palmitate (PNP) was used as a substrate. Briefly, the supernatant of 24 h catechol treated and untreated cells were harvested by centrifugation at 8000 rpm for 10 min. Then, 0.1 mL of culture supernatant was mixed with 900 µL of substrate containing 0.1 mL of substrate A (0.3%PNP) and 0.9 mL of substrate B (consisting 0.2% of sodium deoxycholate and 0.1% of gummi arabicum in 20 mM tris buffer). This reaction mixture was incubated at room temperature for 2 h. After incubation, supernatant was collected and at read at 410 nm.

### Secreted proteinases assay

The effect of catechol in the secretion of proteinase was evaluated by both qualitative and quantitative methods demonstrated by Akcaglar et al., with minor modifications^32^. To qualitatively assess the proteinase secretion, spider medium containing 1% glucose, 0.05% MgSO_4_, 2% agar and 1% of bovine serum albumin (BSA) in the presence and absence of catechol at 64, 128 and 256 µg/mL were prepared. 5 µL of *C. albicans* cells (1 × 10^6^ CFU/mL) was placed on the center of agar plate and incubate at 37° C for 2–3 days. Consecutively, the proteinase production was calculated by measuring the white precipitated zone around colony using Hiantibiotic zone scale (Himedia, Mumbai). In addition, the plates were imaged using gel documentation system (GelDoc XR+, Bio-Rad, United States). To quantify the proteinase production, *C. albicans* was grown in YEPD medium supplemented with catechol at concentration of 64, 128 and 256 µg/mL for 24 h. After incubation, the cell free supernatant was collected through centrifugation at 8000 rpm for 10 min. Then, 0.1 mL of culture supernatant was mixed with citrate buffer consisting 0.2% of BSA. After incubation at room temperature for 10 min, the supernatant was read at 280 nm.

### Cell surface hydrophobicity assay

Initially, *C. albicans* was grown for 24 h with and without catechol at the concentrations of 64, 128 and 256 µg/mL^33^. Then, the cells were resuspended with YEPD medium to obtain an OD of 1.0. Subsequently, 1 mL of toluene (SRL, India) was added to each cell suspensions and vortexed vigorously for 1 min. The tubes were kept at room temperature for 30 min to allow phase separation. Then, the lower aqueous phase was carefully separated and transferred to fresh polystyrene plate. OD was read at 600 nm. The OD value of strain in the YEPD broth without toluene was used as negative control. The relative hydrophobicity index was expressed as adherence to toluene and was calculated using the following formula: [1 − (OD600 nm after vortexing/OD600 nm before vortexing) × 100.

### Preformed biofilm disruption assay

The preformed biofilm disruption ability of catechol was examined using the crystal violet staining method^34^. For preformed biofilms, the overnight *C. albicans* culture was used to inoculate in 24-well MTPs containing spider medium and incubated at 37° C for 48 h. After removal of spent medium, fresh spider broth was added into MTPs along with catechol at varied concentrations (0–1024 µg/mL) and incubated at 37° C for 24 h. After incubation, the biofilm formations were spectroscopically quantified at 570 nm. The percentage of biofilm inhibition was calculated using the above mentioned formula.

### Quantification of *C. albicans* exopolysaccharide

To assess the impact, the catechol on *C. albicans* exopolysaccharide production, phenol-sulphuric acid method was performed^35^. Briefly, *C. albicans* was grown for 24 h in the presence and absence of catechol at the concentration of 64, 128 and 256 µg/mL. After incubation, the cell pellet was obtained through centrifugation at 8000 rpm for 10 min. Then, 1 mL of 0.9% saline was used to resuspend each cell pellet. An equal volume of 5% phenol was added to the suspension, followed by 5 volumes of concentrated sulphuric acid. After incubation at room temperature for 1 h in a dark condition, supernatant was read at 490 nm.

### Extraction of *C. albicans* exopolymeric substances (EPS)

EPS play an inevitable role in rendering resistance to the sessile cells of *C. albicans* against antimicrobial agents and also act like a protective sheath against host immune response^36^. Hence, the influence of catechol in the production of EPS by sessile cells of *C. albicans* was assessed using the previously stated protocol by Badireddy et al.^37^. EPS was extracted from both untreated control and catechol treated biofilm cells. In brief, *C. albicans* cells (1 × 10^6^ CFU/mL) were used to inoculate in YEPD medium supplemented with 10% of FBS in the presence and absence of catechol (at BIC), and incubated at 37° C for 8 h. Subsequent to incubation, the cell-free culture supernatant (CFCS) was separated by centrifugation at 12,000 rpm for 10 min. In order to extract cell bound EPS, the pellet so obtained was suspended in 10 mL of isotonic buffer consisting of 10 mM Tris/HCl pH 8.0, 10 mM EDTA and 2.5% NaCl, and incubated for overnight at 4° C. After incubation, the suspension was subjected to centrifugation at 12,000 rpm for 10 min. The resulting supernatant (cell-bound EPS) was mixed with already collected CFCS. Now, the pooled EPS (both cell-bound and secreted) was precipitated for overnight at − 20° C with double the volume of chilled ethanol. After precipitation, the pelleted form of EPS was obtained by centrifugation for 10 min at 12,000 rpm and stored at 4° C until further quantification.

### Fourier transforms infrared (FTIR) spectroscopic analysis of *C. albicans* EPS

The alteration of EPS components upon treatment with catechol were examined using FTIR (Nicolet iS5 FT-IR Spectrometer, Thermo Scientific, USA)^31^. The extracted EPS was mixed with KBr pellet at the ratio of 1:100 and 100 kg cm^−2^ pressure was applied for 5 min to obtain the pellet from the mixture. Both control and treated cells were scanned in the range of 4000–400 cm^−1^ with 4 cm^−1^ resolution. The obtain KBr pellet spectrum was subtracted from all spectra.

### Extraction and quantification of *C. albicans* ergosterol

The total ergosterol content from *C. albicans* was quantified using the standard method suggested by Arthington-Skaggs et al. with little changes^38^. Briefly, overnight *C. albicans* was used to inoculate 5 mL of YEPD medium with and without catechol (at BIC) and incubated at 37° C for 24 h. Then the culture suspension was centrifuged at 2700 rpm for 5 min to harvest the stationary-phase cells. Then, the cell pellet was dissolved with 300 µL of 25% alcoholic potassium hydroxide solution and vortexed vigorously for 1 min. Next, the tubes were incubated in water bath at 85° C for 1 h. After allowing the tubes to cool at room temperature, 400 µL of mixture containing sterile distilled water and n-heptane (SRL, India) in the ratio of 1: 3 was added to each tube, and subjected to vigorous vortexing for 3 min. Now, the top n-heptane layer entrapping ergosterol was transferred to fresh microfuge tubes and stored at − 20° C for 24 h. Before the spectral analysis, a 100 µL extracted sterol was diluted with five-fold 100% ethanol and then scanned spectrophotometrically between 240 and 300 nm.

### Extraction and FTIR analysis of farnesol

The farnesol was extracted from *C. albicans* using a method demonstrated by Hornby et al. with necessary modifications^39^. In brief, 1 × 10^6^ of *C. albicans* cells was used to inoculate 40 mL of GPP medium (20 g glucose, 6 g Na_2_HPO_4_·7H_2_O, 4 g KH_2_PO_4_, 0.5 g MgS0_4_·7H_2_O, 1 mg CuSO_4_·5H_2_O, 1 mg ZnSO_4_·7H_2_O, 1 mg MnCl_2_, 1 mg FeSO_4_, 20 µg biotin, 200 µg pyridoxine HCl, 200 µg thiamine HCl and 10 mM L-proline) in the presence and absence of catechol (at BIC). Tubes were incubated at 37° C for 24 h under shaking condition. After incubation, the cell suspension was centrifuged at 12,000 rpm for 20 min. Then, the culture supernatant was filter sterilized using 0.2 µm Whatman cellulose nitrate filters affixed in vacuum filtration apparatus. The resulting CFCS was then extracted with 10 mL of ethyl acetate. Dried residues were suspended with 1 mL of 20% ethyl acetate-hexane mixture and transferred to fresh microfuge tubes. Next, the residual solvents were removed under vacuum evaporator. Then, the changes in farnesol production upon treatment with catechol were investigated using FTIR (Nicolet iS5 FT-IR Spectrometer, Thermo Scientific, USA). The extracted farnesol from both catechol treated and untreated cells suspension were mixed with KBr pellet at the ratio of 1:100 and pelleted form of mixture was obtained by applying 100 kg cm^−2^ pressure for 5 min. The pellets were scanned in the range of 4000–400 cm^−1^ with 4 cm^−1^ resolution. The obtain KBr pellet spectrum was subtracted from all spectra.

### Serial-passage experiment

In order to investigate whether repeated exposure of catechol leads to resistance development in *C. albicans*, serial passage experiment was carried out as previously demonstrated by Pierce et al.^40^. Initially, 1 × 10^6^ CFU/mL *C. albicans* cells were used to inoculate 2 mL of YEPD and spider medium with and without catechol at the hyphal inhibitory concentration of 128 µg/mL. Cultures were incubated at 37° C for 24 h with agitation to allow hyphal induction. Every day from then, 20 µL from each culture (both control and catechol treated) were serially transferred into 2 mL of fresh medium (YEPD or spider) supplemented with catechol (at 128 µg/mL). This daily transfer was carried out for 8 days without any interruption and further this was continued for additional 7 days by doubling the concentration of catechol at 256 µg/mL. Before daily transfer, cells were visualized under microscope to examine the hyphal inhibition and also 5 µL of cultures from each YEPD medium (control and treated) were spotted on the YEPD agar plate to investigate the impact of catechol on planktonic growth of *C. albicans*.

### Impact of catechol on antifungal efficacy of azoles and polyenes toward *C. albicans* planktonic cells

Before assessing the potency of catechol in increasing the susceptibility of *C. albicans* towards conventional antifungal drugs, the susceptibility of *C. albicans* against flucanozole, ketoconazole, miconazole, amphotericin-B and nystain were first determined through microbroth dilution assay as explained earlier^25^. The MIC of each antifungal drug against *C. albicans* was determined spectroscopically at OD 600 nm. Next, disk diffusion and micro-broth dilution assays were performed to investigate the efficacy of catechol (at BIC) to enhance the susceptibility of traditional antifungals against *C. albicans*. Briefly, 1 × 10^6^ CFU/mL of *C. albicans* was used to inoculate in YEPD medium with and without catechol and incubated at 37° C for 3 h. In microbroth dilution assay, 1% of untreated control and catechol treated *C. albicans* cells added to the YEPD medium supplemented with appropriated MIC of each anti-fungal drugs. After incubation at 37° C for 24 h, the growth OD was measured at 600 nm using spectrophotometer (Spectra Max 3, Molecular Devices, United States). In disk diffusion assay, both the untreated control and catechol treated cultures were swabbed on separate YEPD agar plates, and antifungal loaded disks were placed on the center of the swabbed agar plates. After incubation at 37° C for 24 h, difference in zone of clearance around the colonies were measured and documented using high resolution CCD camera (GelDoc XR+, Bio-Rad).

### RNA preparation, CDNA synthesis and Real Time PCR analysis (at *C. albicans*)

The differential expression of *C. albicans’* virulence genes upon catechol treatment was evaluated using Real Time PCR (qPCR) analysis^27^. The *C. albicans* cells were grown in the presence and absence of catechol (at 256 µg/mL) for 8 h at 37° C in YEPD broth. Then, the total RNA from the control and catechol treated *C. albicans* cells were extracted using Trizol method and quantified by the nano spectrophotometer (Shimadzu, Japan). Subsequently, cDNA were constructed from the isolated RNA (1 mg/mL) using High capacity cDNA Reverse Transcription kit (Applied Biosystems, USA). qPCR was performed for the three positive (*RAS1*, *HWP1*, *ALS3*) and two negative regulator genes (*NRG1*, *TUP1*) involved in hyphae and biofilm formation. The primers were included individually along with cDNA and SYBR Green kit (Applied Biosystems, USA) at the final reaction volume of 10 µL. The qPCR analysis of selected genes was carried using the thermal cycler (7500 Sequence Detection System). The sequence and functions of the used genes are tabulated in Table. 1. The expression profile of selected genes was normalized using *C. albicans’* ITS region. The fold change in gene expressions was quantified by 2^−ΔΔCT^ method^41^.

**Table 1:** List of the genes, their function and corresponding primer sequence used in the current study.

S. No	Gene	Function	Primer sequence (5′–3′)	
			Forward	Reverse
1	*nrg1*	Negative regulator of transcription	CCAAGTACCTCCACCAGCAT	GGGAGTTGGCCAGTAAATCA
*2*	*tup1*	Negative regulator of transcription	CTTGGAGTTGGCCCATAGAA	TGGTGCCACAATCTGTTGTT
*3*	*hwp1*	Hyphal wall protein Adhesion	GCTCCTGCTCCTGAAATGAC	CTGGAGCAATTGGTGAGGTT
*4*	*ras1*	Cell adhesion, filamentous growth	CCCAACTATTGAGGATTCTTATCGTAAA	TCTCATGGCCAGATATTCTTCTTG
*5*	*als3*	Adhesion, Agglutinin like protein	CAACTTGGGTTATTGAAACAAAAACA	AGAAACAGAAACCCAAGAACAACC

### Statistical analysis

All the experiments were carried out in biological triplicates with at least two experimental replicates and the data were presented as mean ± standard deviation. To evaluate statistical differences between control and treated samples one-way analysis of variance (ANOVA) and Dunnett’s post hoc test was performed using SPSS statistical software 17.0. The significance was represented as *p* ≤ 0.05 and < 0.01, respectively.

## Results and discussion

Over a long period of time, the term drug resistance has been travelling with us owing to the continuous exposure of antimicrobial agents in the fight against infectious diseases. Mechanistically, most of the antifungal drugs administered till date in clinical settings have been tend to render more selective pressure which perpetuates drug resistance. This would lead us to a ‘no drug of last resort’ situation, before which, a promising alternative way to combat drug-resistant pathogens should be evolved. Antivirulence being the best among few alternative therapies, in the present study attempts was made to explore an antivirulence compound from plant resources against drug-resistance *C. albicans*, which cause deadly diseases in immunocompromised patients. As traced to antiquity, plants and plant-derived natural compounds have been playing a pivotal role in medical treatments. In recent days, phytochemicals with their pharmaceutical ability to selectively target the virulence attributes at modest consequences have utterly grabbed the researchers’ attention as an antivirulent agent^42^. Among these phytochemicals, we found catechol, one of the simplest allelochemicals rich in onions, maple and oak, as the promising antivirulence agents targeting biofilm and hyphal formation in *C. albicans*. Despite potent broad-spectrum antimicrobial efficacy against various pathogens^19^, the present study is the first of its kind to layout the inhibitory propensity of catechol at sub-lethal concentrations against different virulence attributes of *C. albicans*. Primarily, a wide range of catechol concentrations were deployed to identify its MIC against *C. albicans* ATCC 10231, and it was determined to be 1024 µg/mL. An earlier report by Kocaçalışkan et al. demonstrated the antimycotic effect of catechol against *Fusarium oxysporum* and *Penicillium italicum*; nevertheless, the data of the present study for the first time unveiled its anticandidal efficacy^18^.

### Catechol at sub-MICs does not affect the basic metabolic function of *C. albicans*

**Figure 1:**
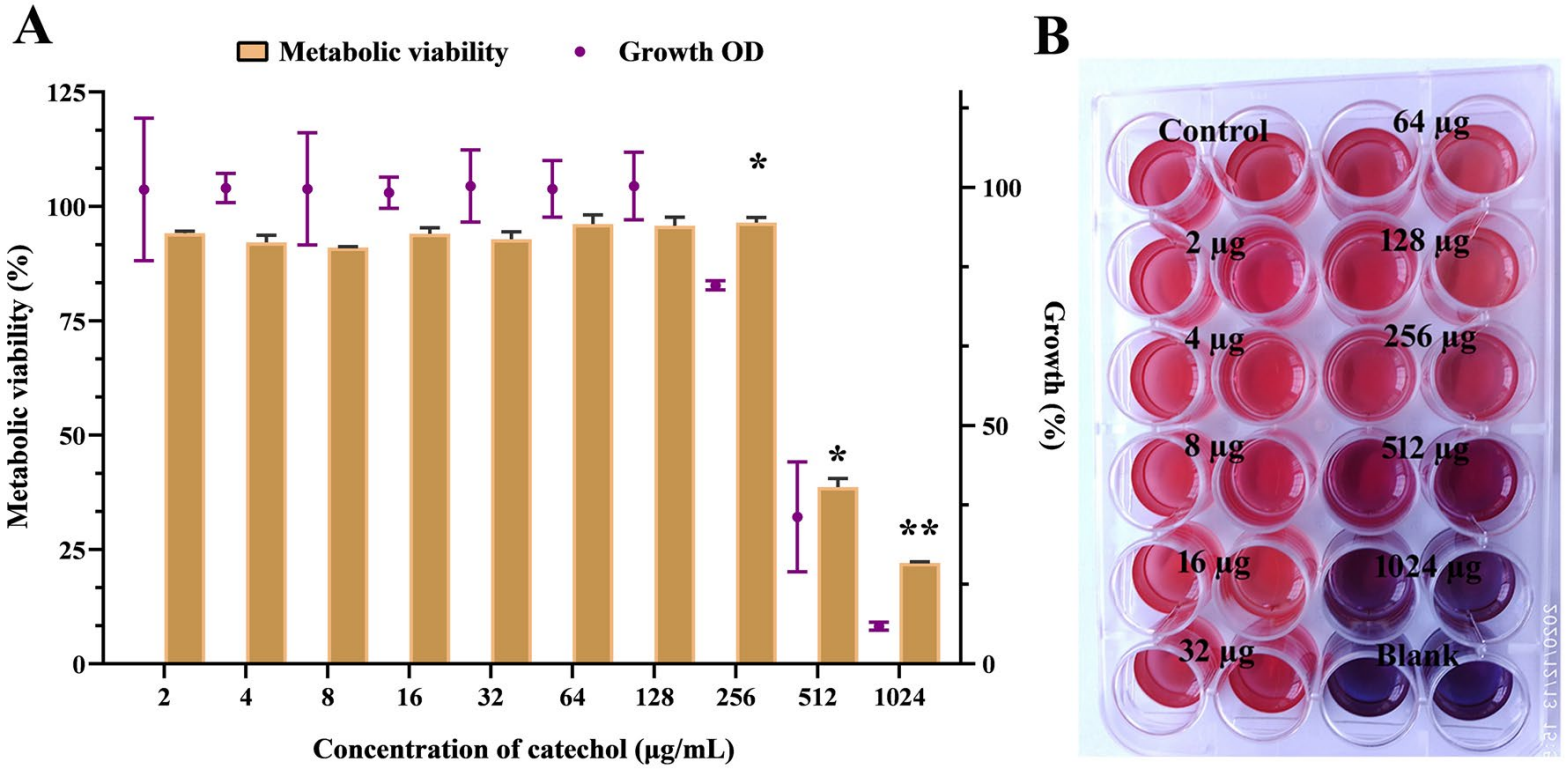
The influence of catechol (0–1024 μg/mL) on *C. albicans* planktonic growth. (**A**) Measurement of *C. albicans* cell density and metabolic viability after grown for 24 h in the presence and absence of catechol using broth micro dilution and alamar blue assay, respectively. Error bars indicates the mean values of three experimental triplicates. The “* “and “**” symbols represents the statistical significance of p < 0.05 and p < 0.01, respectively. (**B**) Representative MTP stained with alamar blue show the true metabolic state of catechol treated *C. albicans* cells.

Since, the fundamental phenomenon of antivirulent is that it should not have any influence on basic metabolism of pathogen, a great deal of attention has been given to evaluate the non-fungicidal effect of catechol at used concentrations against *C. albicans* viability. The obtained result of microbroth dilution assay showed that the sub-MICs of catechol (0–256 µg/mL) did not show any significant impact on the growth of planktonic cells (Fig. 1A). However, the higher concentration of catechol i.e. 1024 µg/mL reduced the *C. albicans* growth to a significant level (p < 0.01). Further, to substantiate the non-fungicidal effect of catechol at sub-MICs, alamar blue assay was performed. Here, an oxidation–reduction (REDOX) indicator resazurin was used to indicate the metabolically active cells. A dose-dependent decrease in the intensity of the pink color or reduced resorufin during catechol treatment was observed, which is depicted in the Fig. 1B. The intensity of pink color produced by the cells exposed with catechol at concentrations up to 256 µg/mL was very close to the intensity of pink color produced by the control cells. However, the retained blue color by the cells exposed with high concentrations (512 and 1024 µg/mL) of catechol showed the un-metabolized state of resazurin, which in turn ascertained high concentration-mediated anticandidal effect of catechol. Taken together, both the growth OD assay and the alamar blue assay confirmed that the catechol at sub-MICs does not exert any negative impact on the growth and viability of *C. albicans* even after 24 h incubation. Hence, below sub-MICs of catechol (0–256 µg/mL) were considered for further analysis to evaluate the antivirulence potential against *C. albicans*.

### Catechol at sub-MIC deteriorates yeast-hyphal transition of *C. albicans*

**Figure 2:**
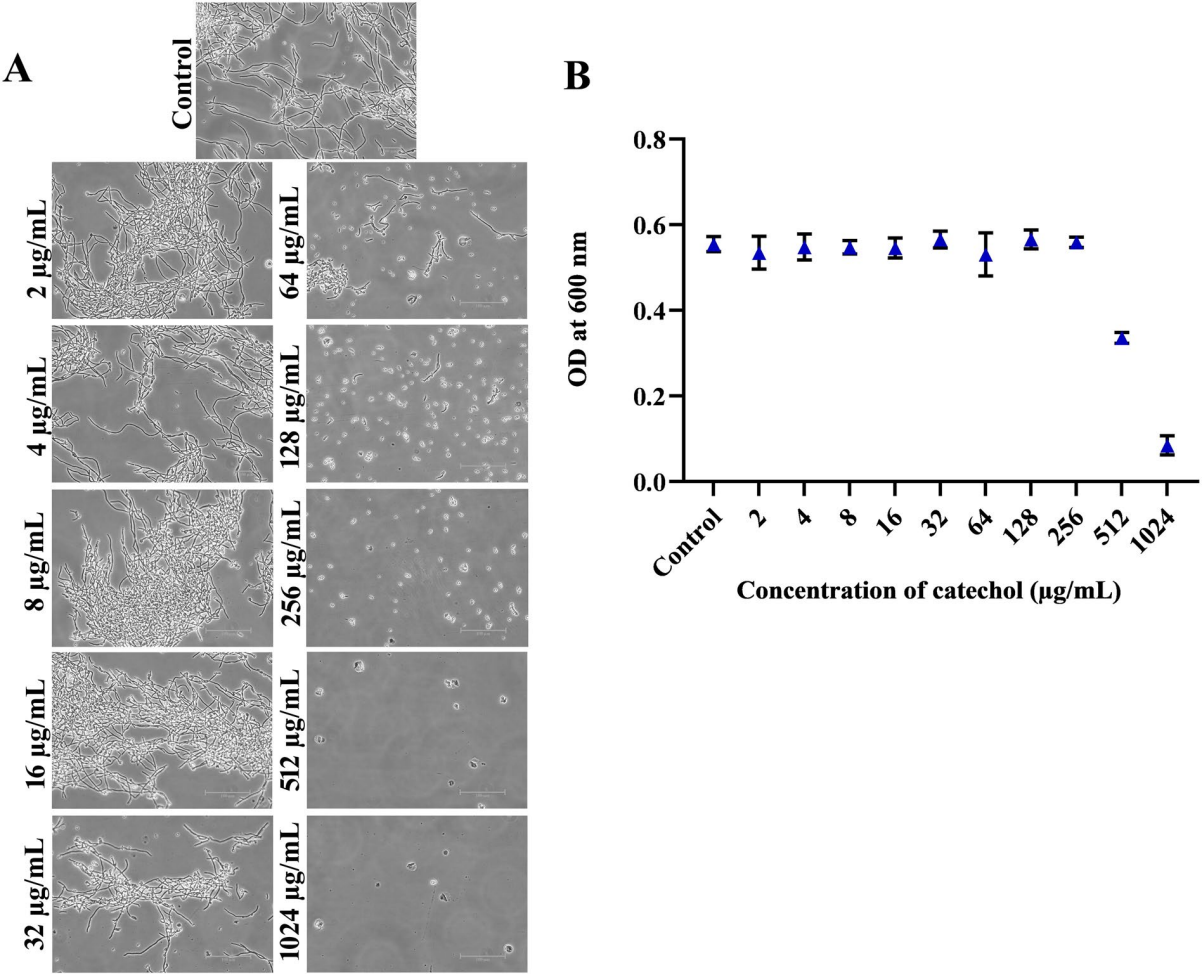
Effect of catechol on yeast to hyphae transition and planktonic growth. (**A**) *C. albicans* was grown on liquid spider medium with and without catechol. After 24 h incubation, test samples were photographed under phase contrast microscope. Micrograph of control group exposed very dense and lengthy filamentous cells, catechol treated groups exposed a more number of evenly distributed yeast cells. (**B**) Impact of catechol at various concentrations on *C. albicans* planktonic growth in spider broth after 24 h incubation.

Pathogenesis of *C. albicans* is attributed to diversified virulence secretions which include, environmental adaptation factors, adhesins, yeast to hyphal transition, secreted enzymes, phenotype switching and biofilm formation^43^. Amongst, yeast to hyphal transition is considered as one of the putative virulence traits, as it coordinately regulates the other virulence traits for host cellular invasion^29^. By placing the *NRG1* gene (a negative regulator of yeast to hyphae transition) into tetracycline promoter for differential regulation of hyphal formation (switch on and off), a research group have testified and ascertained that hyphal form of *C. albicans* is solely responsible to attribute for the mortality during disseminated candidal infections^44^. More other investigators from worldwide have suggested that the molecules with profound efficacy to inhibit yeast to hyphae transition represent an attractive candidate for antivirulence agents^29,34^. Hence, to examine the antihyphal efficacy of catechol at varied concentrations (0–1024 µg/mL), we performed hyphal inhibitory assay in liquid spider medium. When cultured in liquid spider medium, the light micrographs of untreated control groups exposed a dense network of twig-like hyphal formation (Fig. 2). On the other hand, the micrographs of catechol treated groups displayed evenly scattered yeast cells (Fig. 2). Furthermore, the spectroscopic reading of spent spider media also revealed that the catechol does not have any impact on *C. albicans* planktonic growth. This clearly signified the antihyphal efficacy of catechol without posing any negative impact on the metabolic function of both yeast and hyphal cells (Fig. 2). To further analysis the influence of catechol on other virulence traits of *C. albicans*, narrow range of catechol concentration (64, 128, and 256 µg/mL) were used.

### Catechol at sub-MIC inhibits the *C. albicans* hyphal morphology in solid media

**Figure 3:**
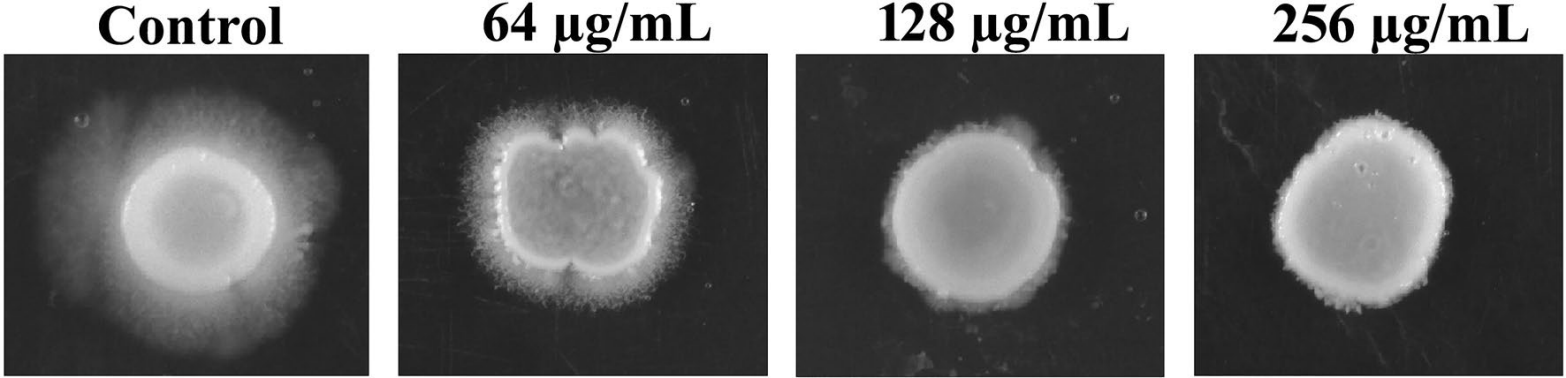
Conformation of yeast to hyphae inhibition by catechol on solid spider media. The fungal colonies were imaged after 5 days of incubation at 37° C. Colonies without catechol were bare the deep hyphal protrusion with irregular cell morphology whereas, colonies treated with catechol displayed a non-filamentous and very smooth topography.

Besides, to know the effect of catechol on *C. albicans* hyphal morphology, spot assay was employed. The overnight culture of *C. albicans* spotted on solid spider medium treated with catechol depicted a smooth colony with profoundly reduced hyphal protrusions at the periphery. On the other hand, the untreated control plates showed the colony characteristics of a typical virulent *C. albicans* i.e. rough colony with dense radius of lengthy hyphal projection (Fig. 3). A gradual reduction in the hyphal protrusion was witnessed with the increasing concentrations of catechol (64, 128 and 256 µg/mL). This is in line with the results of earlier report by Pierce et al. (2015) wherein, structural analogs of diazaspiro-decane exhibited a concentration dependent hyphal inhibitiory efficacy against *C. albicans* hyphal growth. Both the solid and liquid assays data distinctly unveiled the excellent hyphal inhibitory efficacy of catechol^40^.

### Catechol at sub-MIC deconstructs the architecture of *C. albicans* biofilms

**Figure 4:**
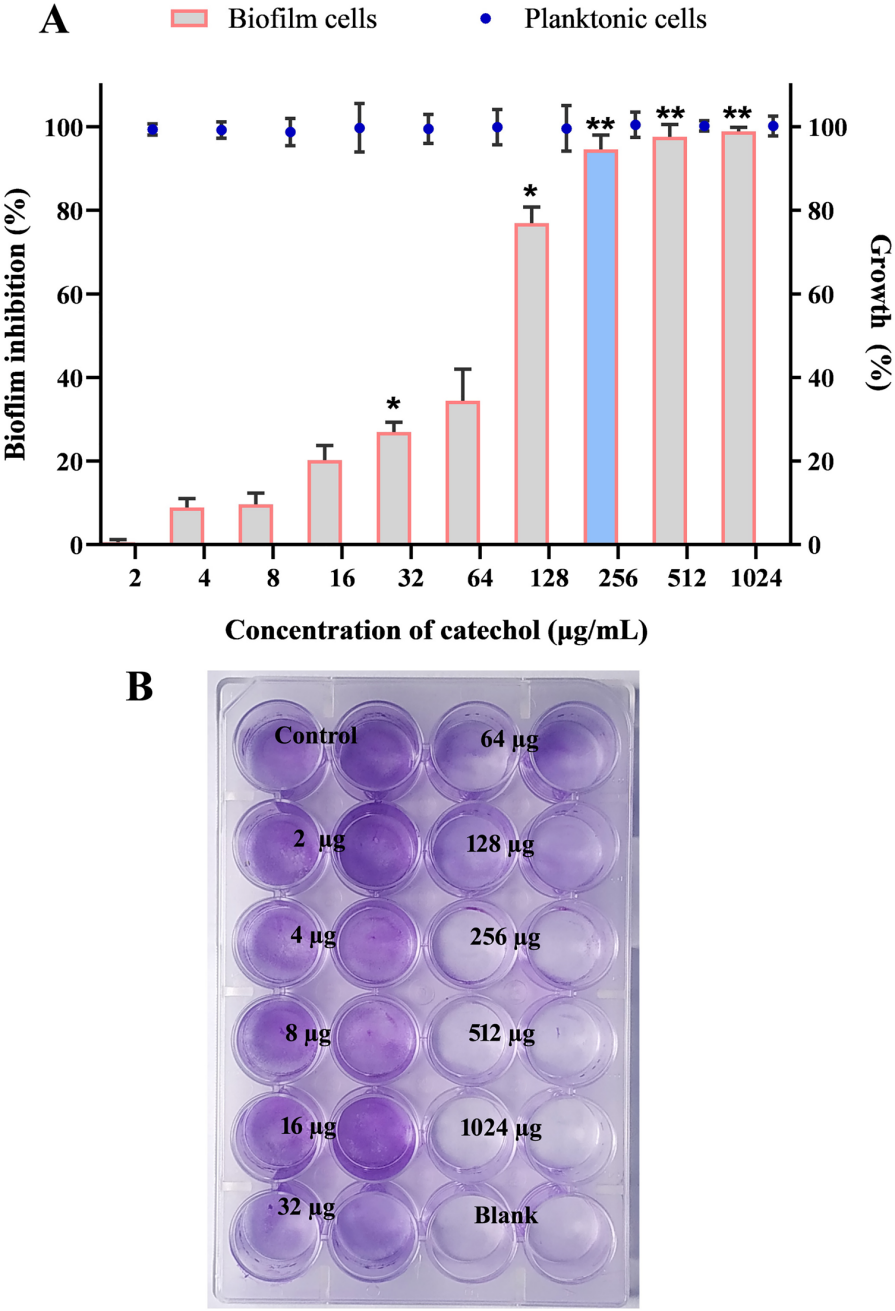
Antibiofilm efficacy of catechol at varied concentrations. (**A**) Measurement of biofilm formation and planktonic growth in presence of catechol at various concentrations. Significant dose dependent reductions of biofilm formation were observed in the cells treated with catechol compared to untreated control without affecting the *C. albicans* planktonic growth. Blue colored bar in the graph signifies the BIC value of catechol. Error bars indicates the mean values of three experimental triplicates. The “* “and “**” symbols represents the statistical significance of p < 0.05 and p < 0.01, respectively. (**B**) Representative MTP image that portraying the impact of catechol on *C. albicans* biofilm.

In general, it is anticipated that the molecules which modulate *C. albicans* yeast to hyphae transition could potentially interrupt the biofilm assemblage owing to the close association between hyphae and biofilms. Further, understanding the clinical significance of biofilm in the emergence of drug resistance, as it renders a safe environment to the residing planktonic cells from the devastating action of antimicrobial agents, the in vitro antibiofilm efficacy of catechol at varied concentrations (0–1024 µg/mL) was assessed by employing crystal violet quantification method^31^. The spectroscopic quantification (at OD 570 nm) of *C. albicans* biofilm biomass grown under a varied range of catechol concentrations (32, 64, 128 and 256 μg/mL) revealed its dose-dependent antibiofilm activity (Fig. 4A). As 256 µg/mL of catechol exhibited a maximum of 93% reduction in *C. albicans* biofilm biomass, this concentration was fixed as BIC. It is also notable that the very low concentration of catechol (32 μg/mL) was effectively control 30% of *C. albicans* biofilms. The representative plate image of biofilm inhibition by catechol was shown in Fig. 4B.

### In situ microscopic examinations authenticate the biofilm obliteration upon catechol treatment

**Figure 5:**
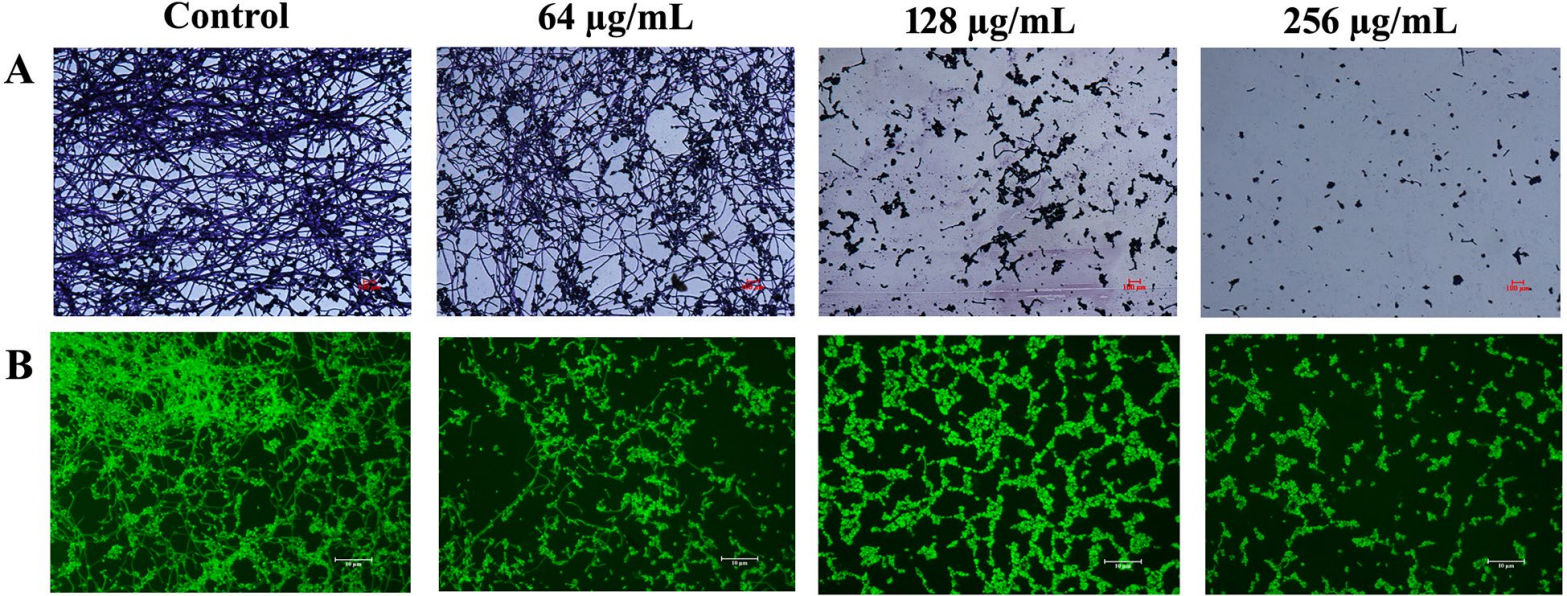
Microscopic visualization of *C. albicans* biofilms formed on the glass surface after 48 h of incubation at 37° C and subsequent staining with 0.1% acridine orange or 0.4% of crystal violet. (**A**) Light (×200 magnification) (**B**) Fluorescence micrograph of control samples showed tightly packed biofilms architectures surrounded with both yeast and hyphal cells. While, the micrograph of catechol treated samples depicted that the considerable decrease in the hyphal and biofilm development (magnification: ×200, scale bar 50 µm or 100 µm).

To comprehensively inspect the catechol mediated biofilm destruction on glass surfaces, light and fluorescence microscopic analyses were performed (Fig. 5). Both the light and fluorescence micrographs of untreated control unveiled a robust crisscross architecture of biofilm encompassing both yeast and long filamentous hyphal cells. In contrast, this characteristic of biofilm architecture was immensely collapsed in a concentration-dependent manner upon 24 h of treatment with catechol. Significant inhibition of micro-colony and hyphal formation was found in *C. albicans* treated with catechol at 256 μg/mL. Likewise, a notable biofilm inhibition was observed at the concentrations of 64 and 128 µg/mL. Microscopic examinations on a whole depicted that catechol attenuates planktonic cells towards forming biofilm architecture in a dose-dependent fashion.

### Catechol at sub-MIC does not kill *C. albicans* sessile cells

**Figure 6:**
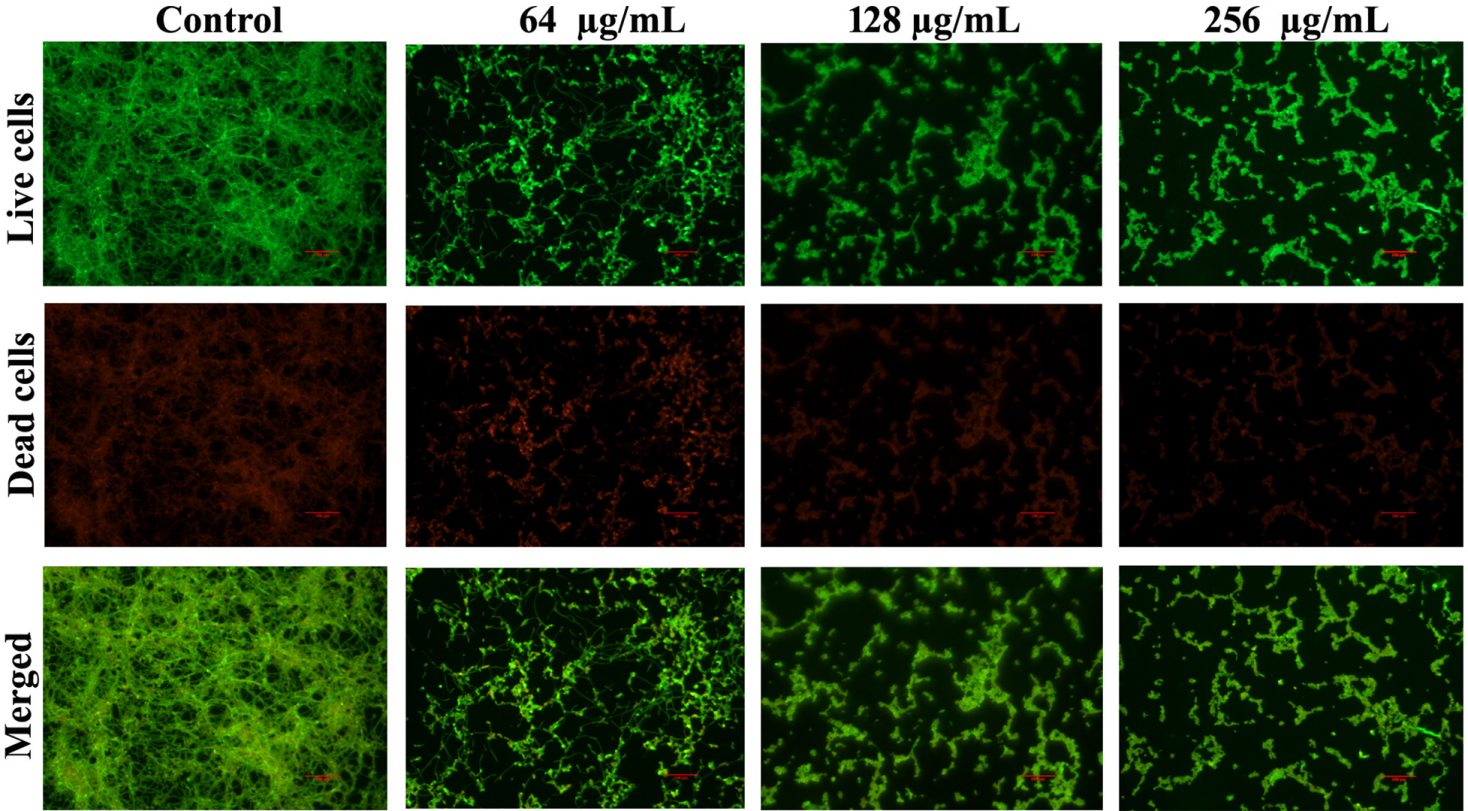
LIVE/DEAD analysis of 48 h catechol treated and untreated *C. albicans* sessile cells. The micrographic images of both catechol treated and untreated control samples reveals the presence of more number of live cells compared to dead cells (magnification: ×200, scale bar 50 µm).

To investigate true viability of *C. albicans* biofilm cells under treatment with catechol, LIVE/ DEAD analysis was performed. AO is a cell permeable dye; hence it can stain the live and dead cells. However, PI can stain the dead cells. As depicted in Fig. 6, there was no obvious difference observed in catechol treated and untreated control samples. The merged microscopic image of control sample revealed the presence of live cells more than the dead cells. Likewise, the cells treated with catechol at the concentration of 64, 128, 256 µg/mL exposed the more number of live cells than dead cells. This assay further affirmed that catechol reduced the *C. albicans* biofilm formation without hampering its growth.

### Catechol at sub-MIC effectively hampers *C. albicans*’ virulence secretions

**Figure 7:**
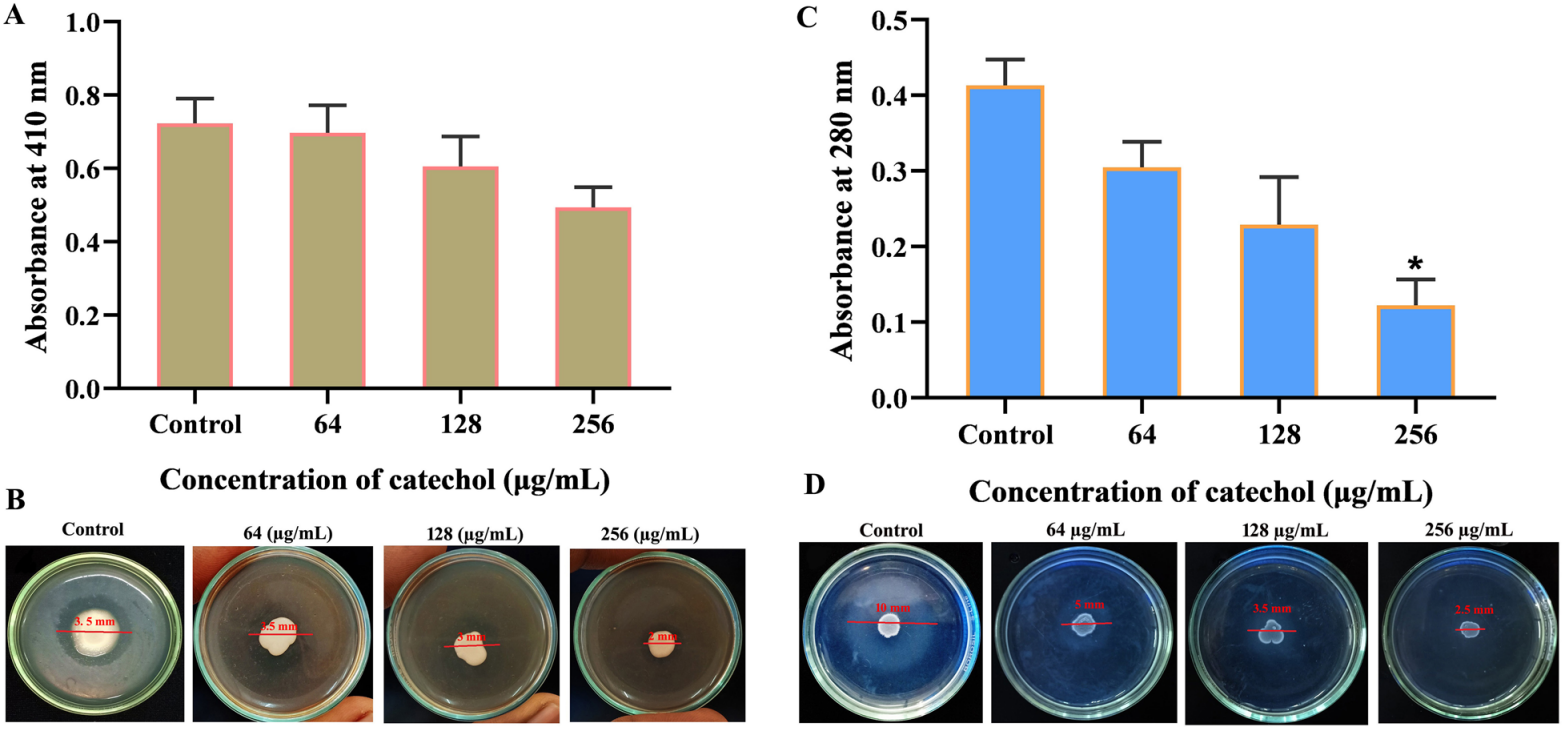
Impact of catechol on virulence enzyme production. (**A**) Quantification of *C. albicans* lipase production after 24 h treatment with catechol at the concentration of 64, 128 and 256 µg/mL. Error bars indicates the mean values of two experimental triplicates. (**B**) *C. albicans* culture was spotted on the plate supplemented with tributyrin in presence and absence of catechol to detect the lipase activity. (**C**) Quantification of *C. albicans* protease production after 24 h treatment with and without catechol (at 64, 128 and 256 µg/mL) using BSA as a substrate. Error bars indicates the two experiments triplicates. (**D**) Qualitative assessment of catechol inhibitory efficacy on the *C. albicans* protease production using BSA plate. Error bars indicates the mean values of two experiments triplicates. The “* “and “**” symbols represents the statistical significance of p < 0.05 and p < 0.01, respectively.

To gain more insight into the antibiofilm and antihyphal action of catechol, differential expression of various virulence traits upon supplementation with catechol was investigated. As depicted earlier, *C. albicans* secretes different extracellular hydrolytic enzymes, which facilitate cellular invasion by proficiently degrading the host’s proteins and cell membrane^45^. Since, secreted aspartyl proteinases (Sap) and lipase of *C. albicans* are being the most discussed extracellular hydrolytic enzymes, the in vitro inhibitory potency of catechol at BIC was assessed against these two virulence enzyme production. Inhibition of *C. albicans* aspartyl proteinase was found to be an attractive strategy to modulate its pathogenesis. For instance, Hoegl et al. (1998) reported that HIV patients who have undergone treatment with HIV protease inhibitors showed resistance to mucosal candidiasis, as the HIV protease inhibitors significantly hampered the production of secretary aspartic proteases from *C. albicans* upon infection^46^. Similarly, it has been reported that compounds that possess the anti-lipase efficacy showed a broad spectrum of antifungal activity against different kinds of pathogenic fungi especially *C. albicans, Cryptococcus neoformans* and *Aspergillus flavus*^47^. Thus, the catechol was taken forward to explore its impact on *C. albicans* virulence scenery such as lipase and protease productions. When *C. albicans* cells were treated with BIC of catechol, both proteolytic and lipolytic zone around the fungal colony were found to be significantly abridged when compared to their respective untreated controls. In protease production assay, white precipitated zone of untreated control plate was found to be 10 mm, whereas, the plates incorporated with varied catechol concentrations 64, 128 and 256 μg/mL displayed the zones of reduced diameter of 5.5, 5 and 3.5 mm, respectively (Fig. 7B). Similarly, the lipolytic transparent zone in lipase assay was found to be 3.5 mm in the control plate. On the other hand, the catechol treated plates at concentrations 64, 128 and 256 μg/mL unveiled a dose-dependent reduction in zone formation i.e. 3.5, 3 and 2 mm, respectively (Fig. 7D). The lipase and protease productions were also quantified using PNP and BSA as substrate, respectively. The data obtained from spectroscopic reading further confirmed that *C. albicans* cells with catechol at the concentrations of 64, 128 and 256 μg/mL showed reduced lipase and protease productions compared to untreated control cells (Fig. 7A,C).

### Catechol treatment potentially diminishes *C. albicans*’ surface hydrophobicity

**Figure 8:**
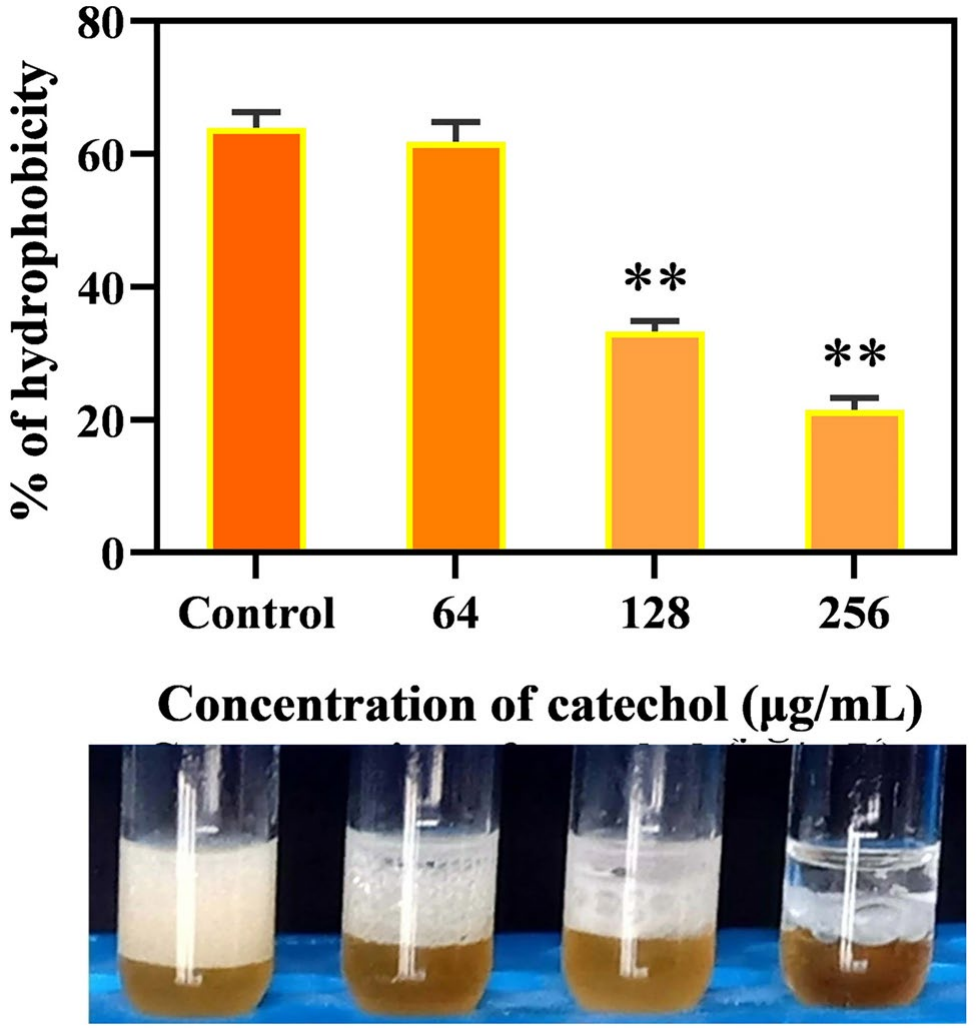
Effect of catechol at its sub-MIC on the *C. albicans* cellular surface hydrophobicity after grown for 24 h. Error bars indicates the mean values of three experimental triplicates. The “* “and “**” symbols represents the statistical significance of p < 0.05 and p < 0.01, respectively.

Cellular hydrophobicity is an important phenomenon as it positively correlates adhesion and biofilm formation of microbial cells and attachment surface (biotic or abiotic), which in sequence increases the virulence of the pathogen^48^. In this current study, the classic MATH assay was performed to evaluate the potency of catechol on the hydrophobic interaction of *C. albicans* with toluene (non-polar solvent) (Fig. 8). After incubation, the hydrophobic index of catechol treated and untreated control cells were evaluated through vortexing with toluene. Phenomenally, catechol has a potent ability to lessen *C. albicans* hydrophobicity in a dose-dependent fashion compared to that of untreated control, which showcases the hydrophobicity index of 63%. Upon treatment with BIC of catechol (256 µg/mL), the hydrophobicity index of *C. albicans* was considerably abridged to about 42%.

### MIC of Catechol disintegrates preformed biofilms of *C. albicans*

**Figure 9:**
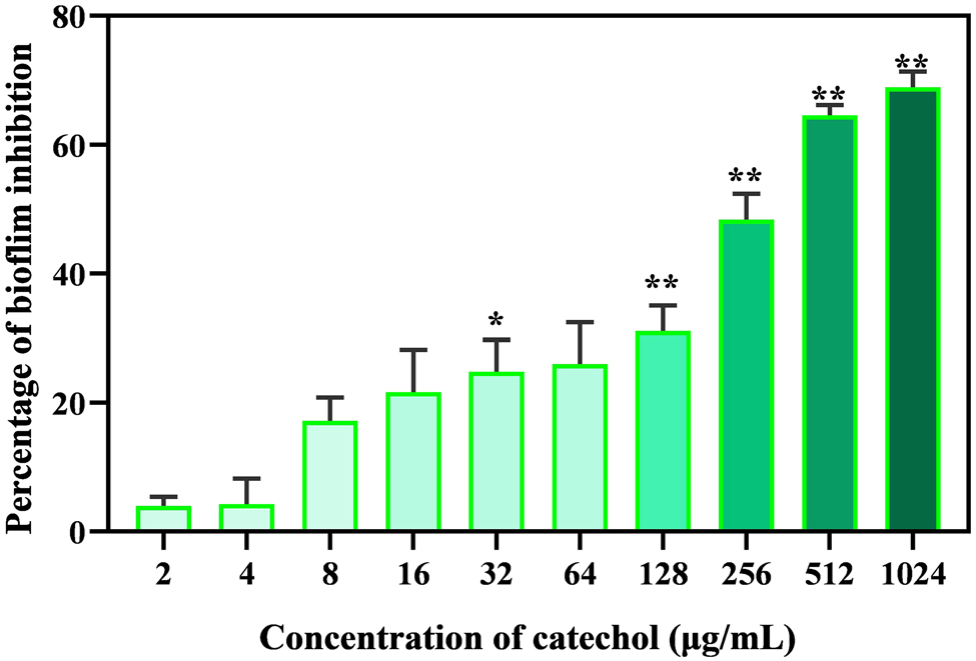
The evaluation of catechol efficacy on preformed biofilm of *C. albicans*. Error bars indicates the mean values of three independent experiments performed in triplicates. The “* “and “**” symbols represents the statistical significance of p < 0.05 and p < 0.01, respectively.

Once the biofilms were established (so called mature biofilm), the embedded sessile cells become tremendously vigorous that makes all kinds of antimicrobial agents to be ineffective. Especially, mature biofilms formed by *C. albicans* are highly heterogeneous in nature in terms of their cellular distribution and extracellular material. As mature biofilms are extremely hard to eradicate with high doses of antimicrobials, it also symbolizes to be a source of recurrent infection^49^. Therefore, the impact of catechol on *C. albicans’* mature biofilm (48 h) was evaluated using crystal violet staining method (Fig. 9). To begin with, the mature biofilm disrupting efficacy of catechol at BIC (256 µg/mL) was evaluated using MTP biofilm biomass assay. Data revealed that BIC of catechol was not sufficient enough to pose any impairment on the preformed biofilms of *C. albicans*. Hence, out of curiosity, we deployed MIC of catechol to treat against 48 h preformed biofilms. As anticipated, the MIC of catechol proficiently disintegrated the preformed biofilm up to about 68%. Hence, it is speculated that the antibiofilm mechanism of catechol (at BIC) could plausibly be due to the hindrance of principal regulators that needed for the initial biofilm assemblage i.e. yeast to hyphal transition and cellular hydrophobicity. Consequently, manifestation with catechol at BIC was not adequate to disrupt and disintegrate the preformed biofilms, as they are highly adhered and enriched with dense network of existing filamentous cells. This result correlates well with the observation by Sivaranjani et al. (2016), wherein BIC of morin was not sufficient to disrupt the preformed biofilms of *Listeria monocytogenes*, however, it exhibited a antibiofilm efficacy through blocking initial attachment^50^.

### Catechol at sub-MIC reduces the *C. albicans* exopolysaccharide production

**Figure 10:**
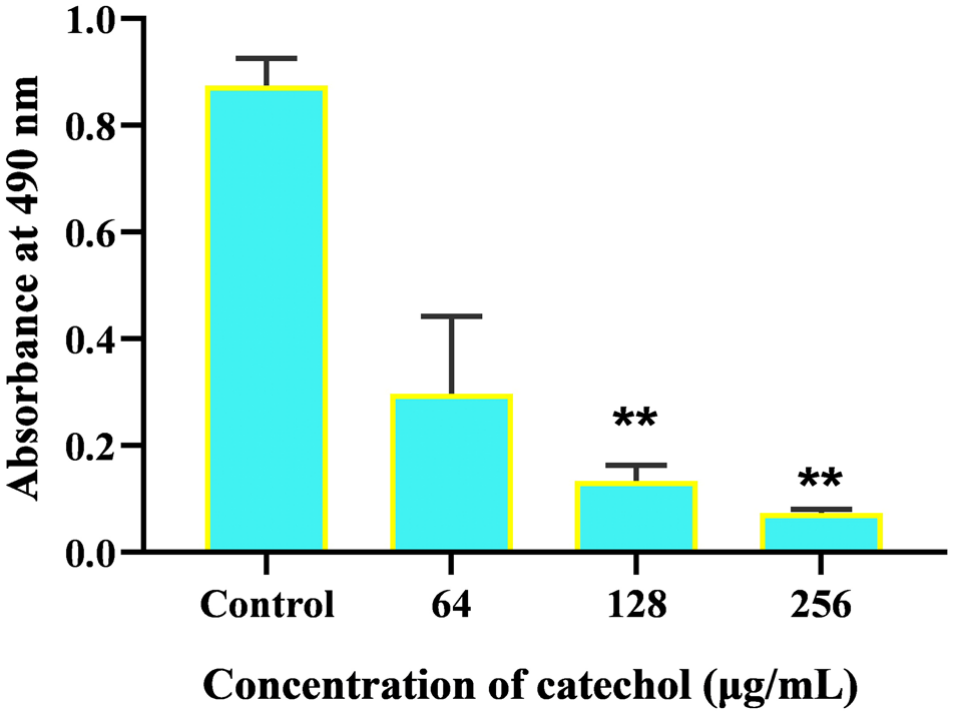
Quantification of total polysaccharide content in *C. albicans* after grown 24 h with and without catechol using phenol-suphuric acid method. Error bars indicates the mean values of two experimental triplicates. The “* “and “**” symbols represents the statistical significance of p < 0.05 and p < 0.01, respectively.

The impact of catechol on the production of exopolysaccharide was quantified by measuring total carbohydrate content by phenol sulfuric acid method. The spectroscopic data revealed that catechol reduced the total carbohydrate content in concentration depended manner (Fig. 10). A significant reduction of carbohydrate content was observed at the catechol concentration of 256 µg/mL (p < 0.01).

### Catechol at BIC mediates alteration in EPS components of *C. albicans*’ biofilms

**Figure 11:**
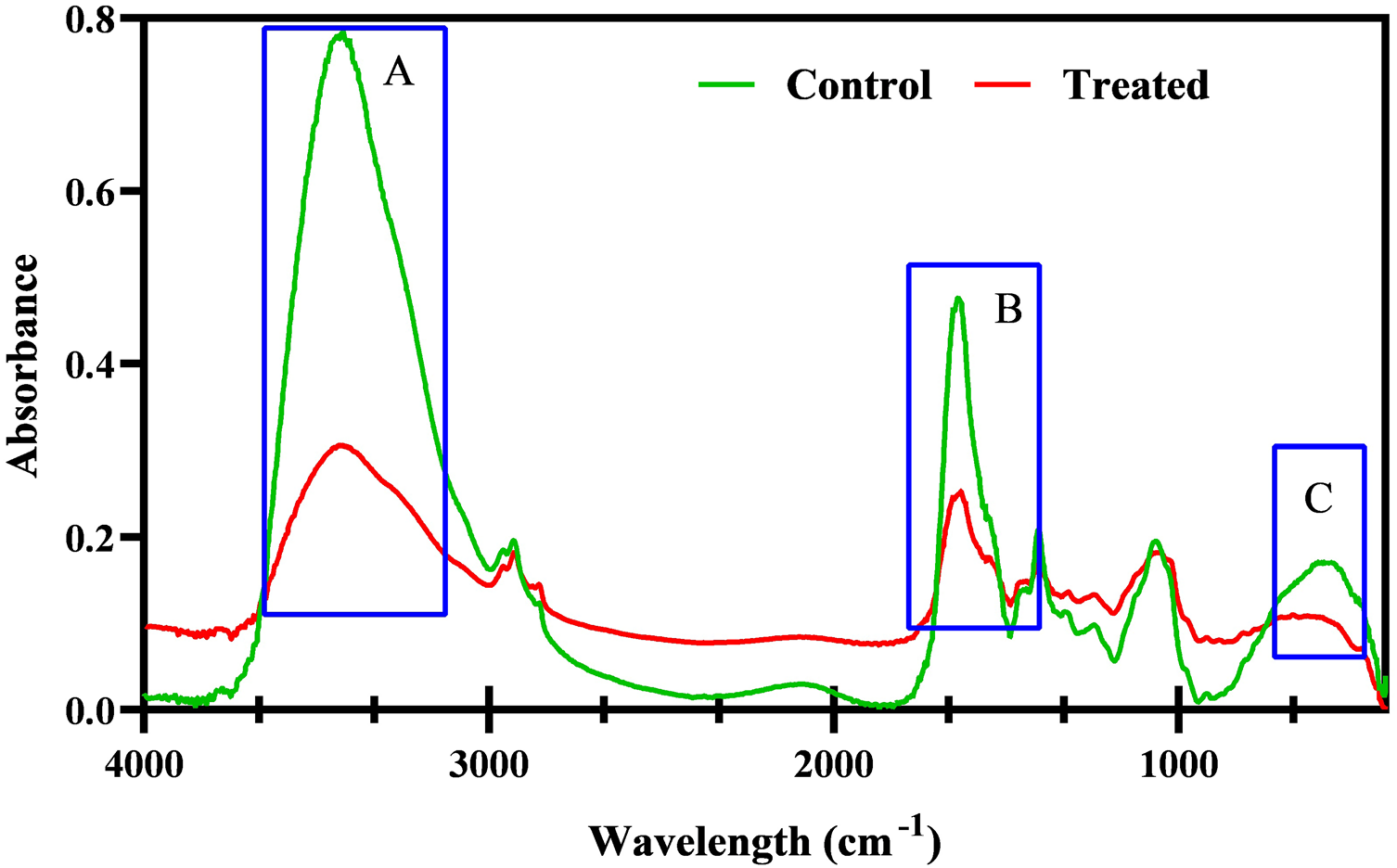
FTIR analysis of *C. albicans EPS*. Spectral region ranging from 4000–400 cm^−1^ represent the control and treated samples. Highlighted region portray the decrease in catechol treated EPS component such as (**a**) amide bonds of peptides (3200–3800 cm − 1), (**b**) proteins (1500–1800 cm^−1^) and (**c**) polysaccharides (200–600 cm^−1^) of microbial cell wall.

The sessile cells are enmeshed in a complex network of hydrated matrix known as EPS, which creates a microenvironment that hinders the penetration of antifungal drugs either through sequestering it or by modulating the degrading enzymes. It is also noted that a maximum of 90% biofilm dry weight accounted for the hydrated EPS matrix, and the remaining portions are occupied by planktonic cells^51^. Therefore, FTIR analysis in the range of 4000 cm^−1^–400 cm^−1^ was performed to understand the catechol-mediated EPS modifications at the biomolecular level, if any. FTIR spectral analysis is a well-known technique commonly used to identify the modifications in EPS components, especially carbohydrates, proteins and lipids. Through the FTIR spectra, various parameters were taken into account to distinguish the cellular compositions of biological samples such as the difference in peak position, bandwidth and band intensity. In the IR spectrum of catechol treated (at 256 µg/mL) sample, the shape and intensity of the absorbance peak were considerably reduced contrast to the spectrum of untreated control. Substantial difference in EPS components under catechol treatment was quite obvious at three spectral regions including (1) 3600–3000 cm^−1^, (21800–1500 cm^−1^ and (3) 800–400 cm^−1^ (Fig. 11) corresponding to the absorptions of lipids, amide bonds of proteins and peptides and polysaccharides, respectively. The catechol mediated decrease in the peak corresponding protein could be attributed to the phenomenal suppression of virulence protein secretion under the presence of catechol. Thus, it is envisaged that catechol could potentially reduce the biofilm assemblage of *C. albicans* by plausibly targeting the pathways driving the synthesis of extracellular proteins and polysaccharides. Further, catechol with its EPS modulating efficacy, it is also anticipated to effectively increase the vulnerability of *C. albicans* towards the active penetration and action of conventional antifungals.

### Manifestation of catechol does not dwindles the ergosterol production in *C. albicans*

**Figure 12:**
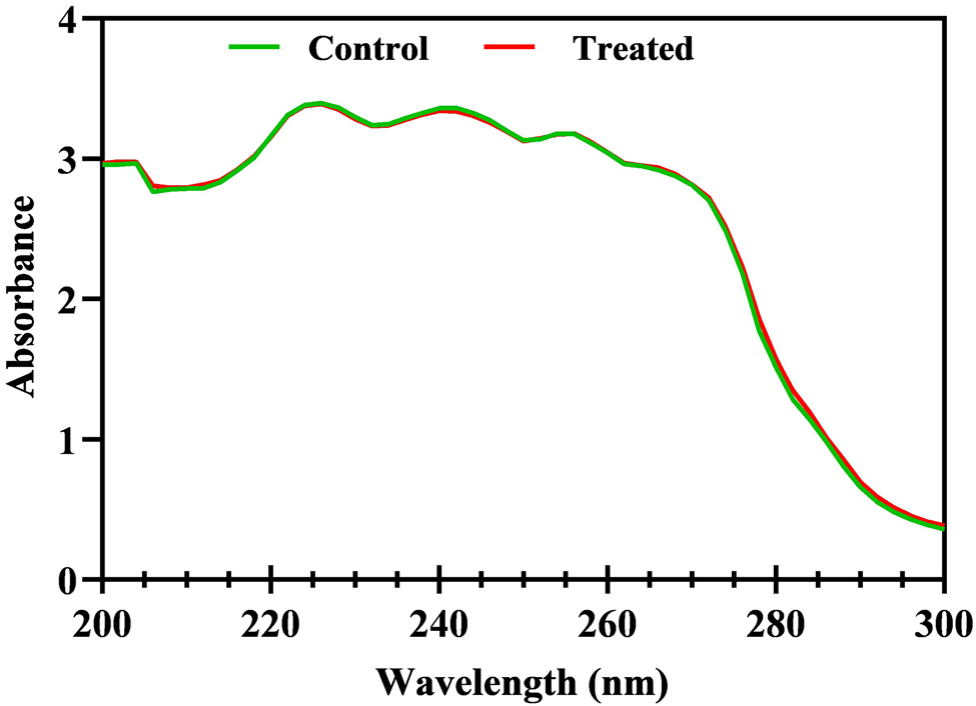
No, influence of catechol (at 256 µg/mL) on *C. albicans* ergosterol production after 24 h treatment.

Ergosterol is the basal component in the fungal cell membrane and plays a crucial role in their permeability and fluidity. Due to its unique function, ergosterol served as a target for most of the conventional antifungal drugs, for instance, polyenes and azoles. The drug resistance to these antifungals has also been reported as they provoked selection pressure by targeting ergosterol^52^. This circumstance demands us to examine the impact of catechol on ergosterol production using UV spectrophotometer. The occurrence of four representative peaks between 240 and 260 nm in UV spectra affirmed the presence of ergosterol and other sterol intermediates in the extracted samples (Fig. 12). The UV spectral profile of control and treated samples displayed peaks of very similar intensity, which proves that the catechol treatment does not pose any impact on the production of ergosterol. On a contrary to our finding, the treatment with antivirulence drug combinations (quinic acid and undecanoic acid) reduced the production of ergosterol as demonstrated in the report by muthamil et al.^31^. Since, the ergosterol biosynthetic pathway is not being targeted by catechol, it is appropriate to state that catechol would not mediate resistance development.

### Catechol triggers the production of farnesol –the quorum sensing molecule of *C. albicans*

**Figure 13:**
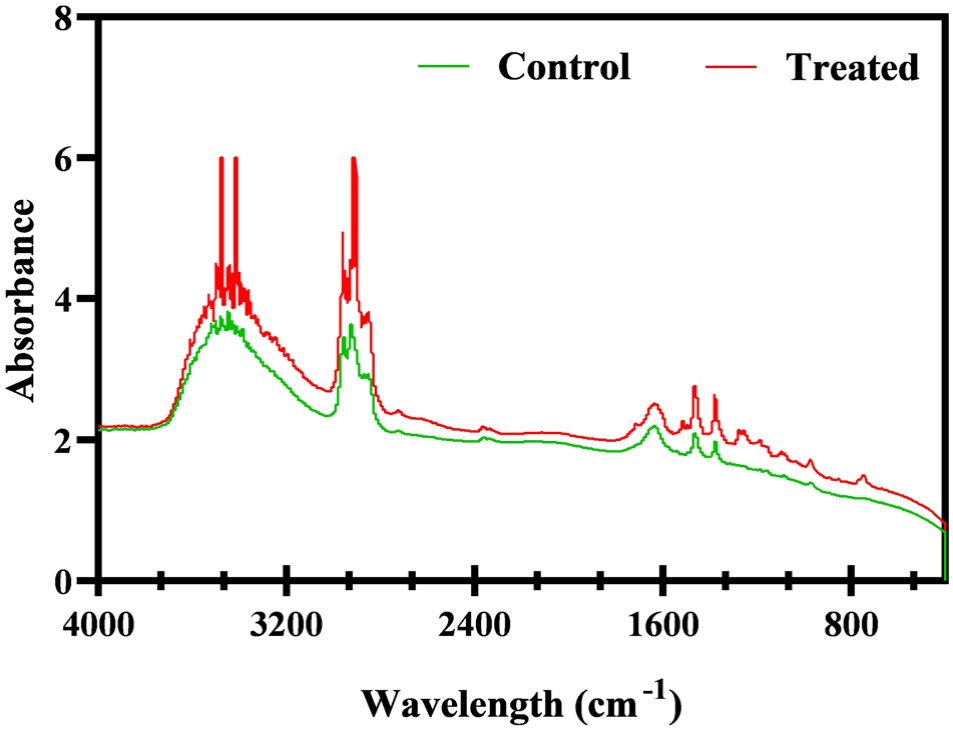
FTIR analysis of *C. albicans* farnesol upon 24 h treatment with catechol (at 256 µg/mL).

The hyphal formation in *C. albicans* necessarily involves two important pathways namely, Ras-dependent (Ras-cAMP-PKA) and Ras-independent (Ubr1-Cup9).As far as the pathogenesis and infection by *C. albicans* are concerned, yeast to hyphal transition has been mediated through the Ras-cAMP-PKA pathway^53^. In *C. albicans*, farnesol is the quorum sensing molecule*,* which naturally blocks the yeast to hyphae transition at relatively higher concentrations. At high cell density, the increased farnesol production negatively regulates Ras-cAMP-PKA signaling pathway by attenuating the Ras1 binding towards Ras associated domain of adenylate cyclase (cyr1) [positive regulator of yeast to hyphal switching]^54^. In order to affirm that the observed antihyphal activity might be due to increased farnesol production, FTIR analysis was performed (Fig. 13). The detected peaks were compared with the FTIR library search to confirm the presence of farnesol. The obtained FTIR spectral profile certainly showcases that the absorbance peak of treated was two-fold higher than the absorbance peak of control. Thus, it is envisaged that the mechanism of antihyphal action of catechol could be the activation of quorum sensing molecule in *C. albicans* i.e. farnesol. In par with this, a study by Hornby and Nickerson (2004) demonstrated a farnesol-induced antifungal efficacy of azole drugs against *C. albicans*; however, the current study claims for farnesol-induced antihyphal efficacy of a phytochemical^39^.

### *C. albicans* does not exerts resistant menace against catechol’s repeated exposure

**Figure 14:**
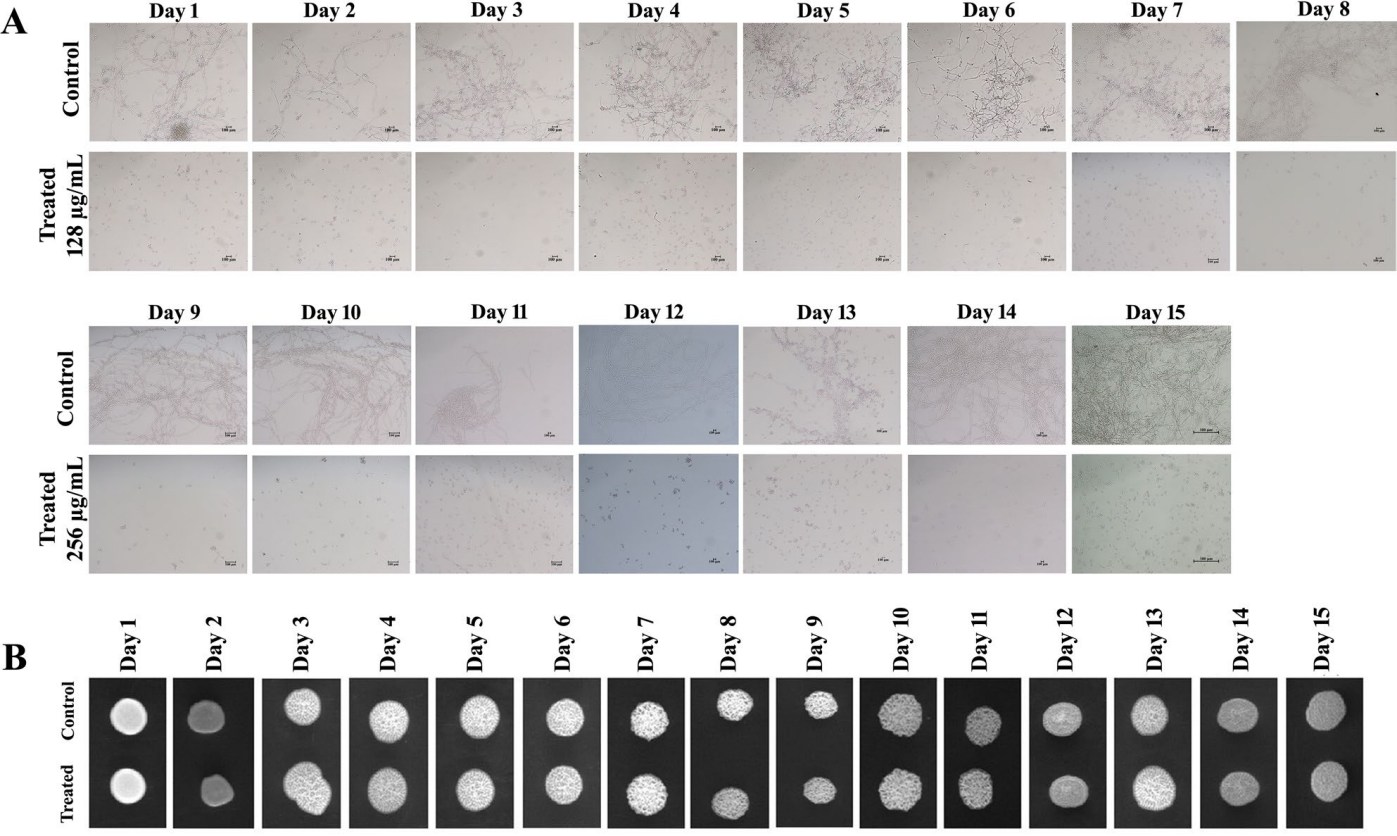
Serial passage experiment by repeated exposure of catechol to *C. albicans* cells on both YEPD and spider liquid media. (**A**) Light graphs were displayed the potential of catechol in inhibiting the yeast to hyphae transition even after serial 15 passages (magnification ×200, scale bar 100 μm). (**B**) Spot assay revealed non-impairment of *C. albicans* metabolic growth in presence and absence of catechol even after 15 serial passages.

As described earlier, drug-resistant strains have been constantly increasing owing to the continuous exposure of antimicrobials, which lead to cause conflicting results compared with susceptible strains^15^. This issue guides us to investigate whether continuous exposure with catechol would induce the development of resistance in *C. albicans*. Hence, the serial passage experiment was performed to evaluate the possibility of spontaneous resistance development upon continuous exposure with catechol as previously demonstrated by Pierce et al.^40^. Initially, *C. albicans* populations were exposed to catechol at its hyphal inhibitory concentration (128 μg/mL) for 8 days in both YEPD and spider medium. Then, the concentration of catechol was increased up to twofold (256 μg/mL) for further 7 days. The populations in the spider medium were continuously monitored for hyphal inhibition under the light microscope every day (Fig. 14A). The micrographs of untreated control cells bare the deep and elongated hyphal formation. As expected, the catechol-treated *C. albicans* cells displayed evenly distributed yeast cells even after 15 days of serial passage. These results evidently demonstrated the long-lasting anti-hyphal efficacy of catechol under subsequent treatment for 15 days. Besides, the cells treated with catechol in the YEPD medium were spotted on the YEPD agar plates to investigate the impact of catechol on the metabolic viability of *C. albicans*. The obtained results revealed that the prolonged catechol manifestation did not display any marked effect on the growth of *C. albicans* (Fig. 14B). Thus, the serial passage data reinforces that catechol-mediated antivirulence therapy does not induce the spontaneous emergence of resistance.

### Pretreatment with catechol potentiates the antifungal efficacy of azoles and polyenes

**Figure 15:**
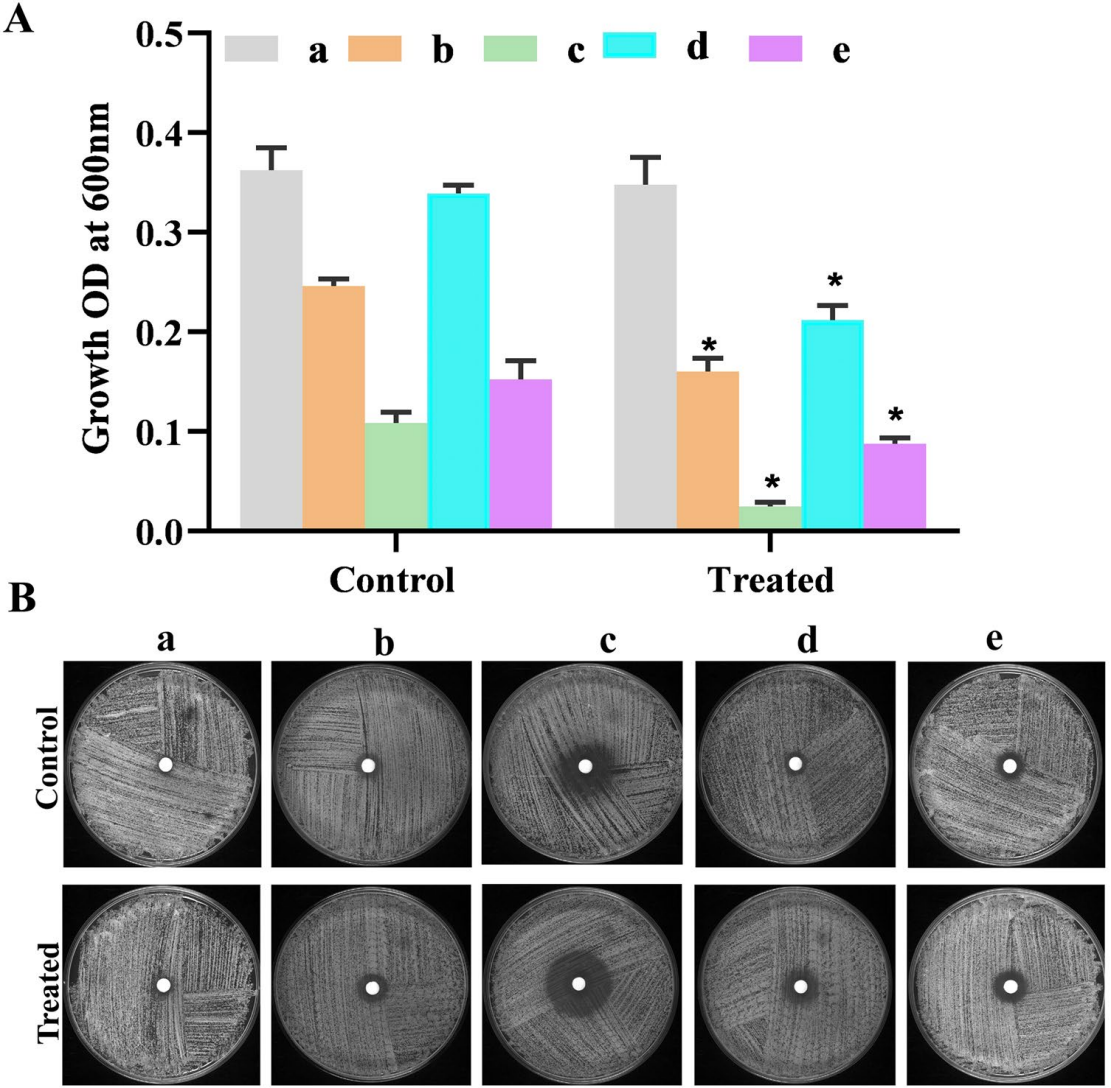
(**A**) Antifungal susceptibility testing of catechol pretreated (3 h) *C. albicans* cells after 24 h incubation using broth micro dilution assay. (**B**) Antifungal susceptibility testing of catechol pretreated *C. albicans* cells to conventional antifungals using disc diffusion method. (a) Flucanozole (b) micanozole (c) ketoconazole (d) nystain and (e) amphotericin-B. Error bars indicates the mean values of three experimental duplicates. The “* “and “**” symbols represents the statistical significance of p < 0.05 and p < 0.01, respectively.

Combination therapy is one of the effective approaches for the reconsideration of old drugs that are unavailable in medical settings today. Most of the synergistic studies have deliberated that the utilization of antibiofilm agents in combination with antifungal agents would potentially sensitizes the pathogen to antifungal agents^55^. Moreover, recent reports have also illustrated that a combination of antifungal agents with newly identified phytochemicals exhibited more efficacy with low toxicity and a broader spectrum of action^2^. For example, a research group identified that the combined action of eugenol significantly reduced the SMIC (sessile MICs) of fluconazole to 32-fold^56^. Similarly, quite a few investigators observed that the synergistic therapy by the antibiofilm agents such as chloroquine and cyclosporine also potentiate the susceptibility rate of *C. albicans* to conventional antifungals^57^. In this milieu, we further examined the efficacy of catechol in potentiating the anti-candidal activity of known conventional antifungal drugs using microbroth dilution and disc diffusion assay. Primarily, the MICs of used antifungals viz., fluconazole, amphotericin-B, ketoconazole, miconazole and nystatin were identified to be 256, 3, 16, 16 and 4 μg/mL, respectively. As anticipated, the data of microbroth dilution also exposed that pretreatment with catechol has reduced the growth OD compared to untreated control *C. albicans* cells (Fig. 15A). Furthermore, the results of the Kirby-Bauer disc diffusion susceptibility test revealed that *C. albicans* pretreatment with catechol (128 μg/mL) for 12 h displayed an increased zone of clearance around the discs (loaded with amphotericin-B, ketoconazole, miconazole and nystatin) compared to that of the colony formed by untreated *C. albicans* (Fig. 15B). However, catechol did not exhibit even a marginal effect over the action of fluconazole. These data signify that the catechol beyond mitigating the hyphal and biofilm formation, it also potentiates the susceptibility of *C. albicans* towards antifungals to a greater extent, which is an excellent pharmaceutical characteristic feature exhibited by a molecule to be an effective alternative to antibiotics.

#### Catechol treatment down-regulates the candidate genes of Ras-cAMP-PKA pathway

**Figure 16:**
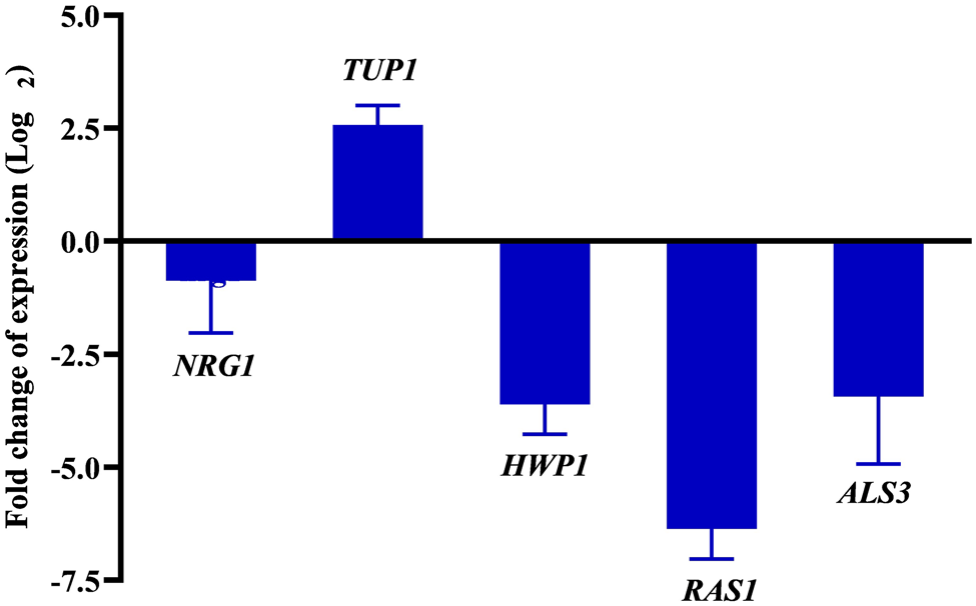
Expression profiles of used candidate genes at transcriptomic level after treatment with catechol at 37° C for 8 h. The genes responsible for adhesion, filamentous growth and biofilm formation such as *HWP1*, *RAS1*, and *ALS3* were significantly downregulated in catechol treated cells. *TUP1*, a negative transcription regulator of filamentation was upregulated. The relative gene expression was calculated by 2^−ΔΔCT^ method using *C. albicans* ITS region as housekeeping internal control gene. Error bars indicates the mean values of three experimental duplicates. The “* “and “**” symbols represents the statistical significance of p < 0.05 and p < 0.01, respectively.

To further evaluate the antihyphal and antibiofilm effect of catechol at transcriptomic level, qPCR analysis was performed. As previously discussed, Ras-cAMP-PKA pathway was found to play a vital role than the all other signal transduction pathways in *C. albicans* pathogenesis^53^. In this pathway, *RAS1* act as positive regulator binds with *CYR1* (adenylate cyclase) and thereby regulates the expression of several virulence genes responsible for filamentation and host cell invasion. On the other hand, *NRG1* negatively regulates the hyphal initiation along with the co-repressor *TUP1*^54^. With this background, we have selected five candidate genes such as *NRG1*, *TUP1*, *HWP1*, *ALS3* and *RAS1* involved in Ras-cAMP-PKA pathway. As anticipated, the qPCR data unveiled the down-regulation of hyphae specific genes in the presence of catechol (at BIC). *RAS1* is an upstream regulator gene of Ras-cAMP-PKA pathway found to be downregulated upto − 6.3 fold during catechol treatment (Fig. 16). On account of this, the other two downstream activating genes such as *HWP1* and *ALS3* were also downregulated to − 3.6 and − 3.4 fold, respectively.

#### Catechol triggered farnesol-mediated hyphal inhibition through up-regulation of *TUP1*

In addition, one of the negative regulator genes *TUP1* was found to be significantly upregulated upto 2.5 fold (p < 0.05). *TUP1* was reported to negatively regulate the genes accountable for initiating filamentous growth by interacting with co-repressor *NRG1*. A study by Kebaara et al. (2008) demonstrated the correlation between the farnesol mediated hyphal inhibition and increased expression of *TUP1* mRNA in *C. albicans*^58^. They have proved that the addition of farnesol leads to increase in *TUP1* expression at both transcriptional and translational level, and also found that farnesol was unable to inhibit filamentation in *TUP1* mutant stains. In comparison with the previous finding, we have suggested that the mode of action of catechol could be plausibly by triggering the quorum sensing molecule—farnesol, which subsequently inhibited the hyphal and biofilm formation in *C. albicans*.

**Figure 17:**
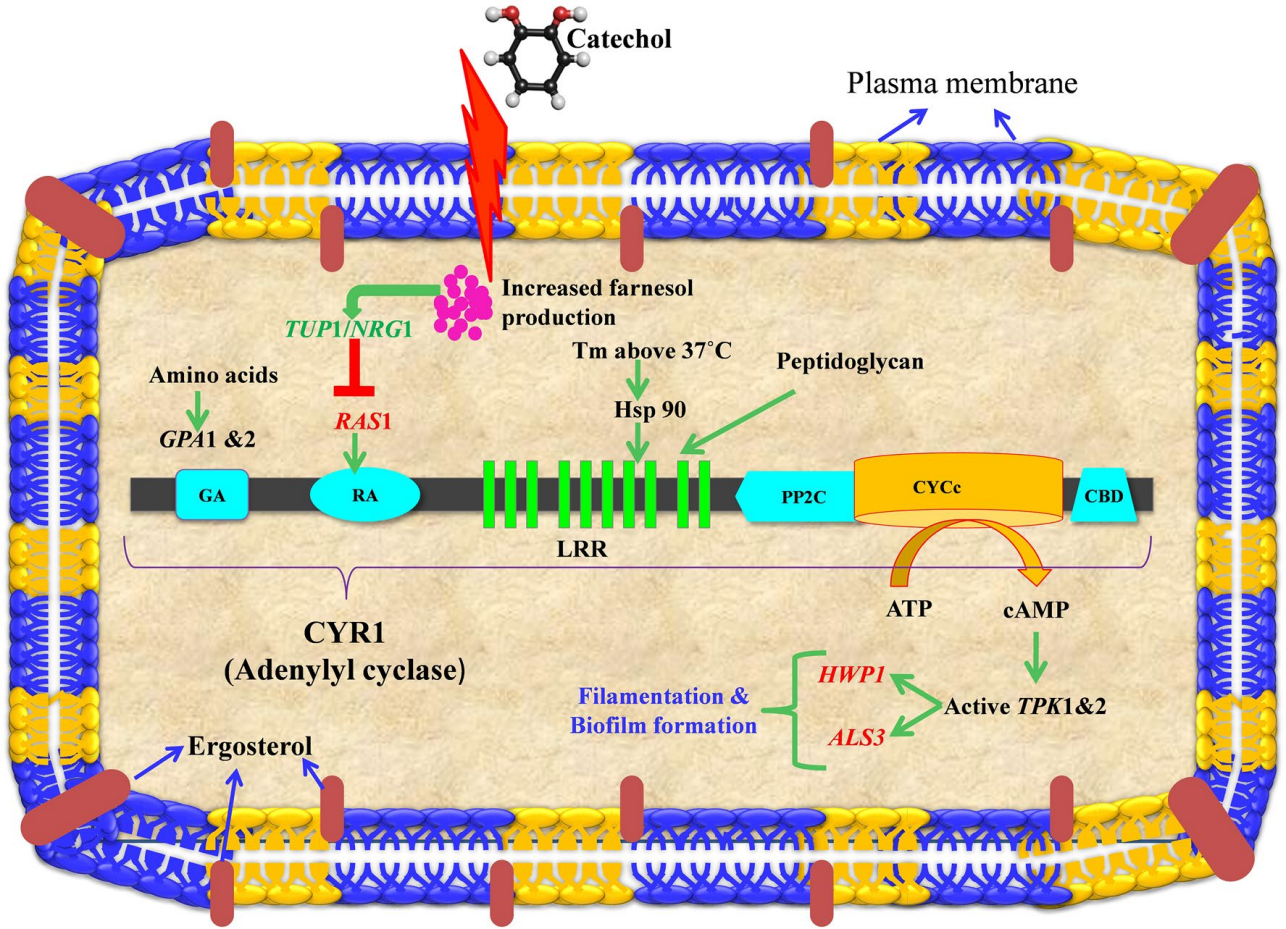
Schematic illustration of differential expression of genes involved in *C. albicans* Ras-cAMP-PKA pathway during catechol exposure. Unregulated and down regulated genes were indicated using red and green colored arrow, respectively.

However, unlike *TUP1*, the expression profile of another negative regulator *NRG1* was downregulated, but not at the significant level (− 0.87 fold). Studies on molecular pathogenesis of *C. albicans* have demonstrated the disappearance of *NRG1* from hyphae specific promoters during the initial 30 min of hyphal induction^54^. Within 60 min of time the level of *NRG1* becomes normal. This could be attributed to the weak fold change in *NRG1* expression between catechol treated and untreated control cells. The overall gene expression profile of C. albicans genes under catechol treatment was illustrated in Fig. 17.

## Conclusions

The use of an antivirulent agent, especially plant-derived phytochemicals as alternative to antimicrobial chemotherapy would be an effective measure to control clinically important fungal pathogens. The current research work illustrates the antibiofilm and antihyphal efficiencies of catechol against a predominant fungal pathogen *C. albicans*. Moreover, inferred data divulge that catechol is successful in substantially reducing the secretary virulence enzymes viz., lipase and protease. Besides, the study also confirms that treatment with catechol does not compromise any fundamental biosynthetic components of *C. albicans* specifically ergosterol. Further, it is envisaged that the antivirulence mode of action of catechol against *C. albicans* could be the triggering of quorum sensing molecule—farnesol, whose accumulation naturally hampers hyphal elongation and its prerequisite—biofilm formation through increasing the expression of *TUP1* (hyphal negative regulator). The serial passage experiment further reinforced the sustained non-fungicidal—antihyphal efficacy of catechol for a long period of time. Although, the present study has well demonstrated yet another pharmacological dimension of catechol against the virulence of *C. albicans* (ATCC 10231), further in vitro and in vivo studies are warranted to authenticate its antivirulence efficacy on clinical isolates of *C. albicans* as well. Overall, the present study provides new insights for the use of a natural compound—catechol as an alternative remedy to treat drug-resistant *C. albicans* infections.
