# Author Correction: Catechol thwarts virulent dimorphism in *Candida albicans* and potentiates the antifungal efficacy of azoles and polyenes

**DOI:** 10.1038/s41598-021-01925-9

**Published:** 2021-11-15

**Authors:** Ravi Jothi, Ravichellam Sangavi, Ponnuchamy Kumar, Shunmugiah Karutha Pandian, Shanmugaraj Gowrishankar

**Affiliations:** 1 Department of Biotechnology, Science Campus, Alagappa University, Karaikudi, Tamil Nadu 630 003 India; 2 Food Chemistry and Molecular Cancer Biology Lab, Department of Animal Health and Management, Alagappa University, Karaikudi, India

Correction to: *Scientific Reports* https://doi.org/10.1038/s41598-021-00485-2, published online 26 October 2021

The original version of this Article contained errors in the spelling of the authors Ravi Jothi, Ravichellam Sangavi, Ponnuchamy Kumar, Shunmugiah Karutha Pandian & Shanmugaraj Gowrishankar which were incorrectly given as Jothi Ravi, Sangavi Ravichellam, Kumar Ponnuchamy, Karutha Pandian Shunmugiah & Gowrishankar Shanmugaraj respectively.

The original Article has been corrected.

